# Role of ecology in shaping external nasal morphology in bats and implications for olfactory tracking

**DOI:** 10.1371/journal.pone.0226689

**Published:** 2020-01-08

**Authors:** Alyson F. Brokaw, Michael Smotherman

**Affiliations:** 1 Interdisciplinary Program in Ecology and Evolutionary Biology, Texas A&M University, College Station, Texas, United States of America; 2 Department of Biology, Texas A&M University, College Station, Texas, United States of America; University of Western Ontario, CANADA

## Abstract

Many animals display morphological adaptations of the nose that improve their ability to detect and track odors. Bilateral odor sampling improves an animals’ ability to navigate using olfaction and increased separation of the nostrils facilitates olfactory source localization. Many bats use odors to find food and mates and bats display an elaborate diversity of facial features. Prior studies have quantified how variations in facial features correlate with echolocation and feeding ecology, but surprisingly none have asked whether bat noses might be adapted for olfactory tracking in flight. We predicted that bat species that rely upon odor cues while foraging would have greater nostril separation in support of olfactory tropotaxis. Using museum specimens, we measured the external nose and cranial morphology of 40 New World bat species. Diet had a significant effect on external nose morphology, but contrary to our predictions, insectivorous bats had the largest relative separation of nostrils, while nectar feeding species had the narrowest nostril widths. Furthermore, nasal echolocating bats had significantly narrower nostrils than oral emitting bats, reflecting a potential trade-off between sonar pulse emission and stereo-olfaction in those species. To our knowledge, this is the first study to evaluate the evolutionary interactions between olfaction and echolocation in shaping the external morphology of a facial feature using modern phylogenetic comparative methods. Future work pairing olfactory morphology with tracking behavior will provide more insight into how animals such as bats integrate olfactory information while foraging.

## Introduction

Animals rely on chemical signals to detect, identify, discriminate, and localize the resources critical for their survival and fitness, including food, shelter, and mates. Tracking an odor to its source (localization) is a complex task, integrating the internal characteristics of an organism (such as nasal anatomy, receptor physiology, central sensory integration circuits, locomotion patterns, *etc*) with the physical characteristics of the chemical odor and the surrounding environment [1]. Odors move through the environment in complex, discontinuous, and variable odor plumes, presenting a complex environment in which animals must rely on various algorithms or strategies in which to extract and use odor information from the environment.

Animals also display diverse behavioral responses and strategies for following an odor trail to its source vary with habitat, size and locomotor speeds. Olfactory klinotaxis (or true gradient search) is movement through an olfactory gradient with successive sampling at different locations [2]. To be effective, this strategy requires close proximity to the odor source, since at farther distances turbulence and advection begin to create patchier distributions of odor concentrations. Olfactory tropotaxis is the ability to simultaneously compare odor inputs among multiple receptors, such as antennae or nostrils [2]. Animals can use tropotactic mechanisms to orient towards an odor based on concentration gradient [3] or time of odor arrival [4] Bilateral processing of odors (stereo-olfaction) is crucial in the olfactory localization behavior of a wide range of taxa, including insects [5,6], mollusks [7,8], crustaceans [9], fish [4], and mammals [10–13]. Bilateral odor sampling is necessary for rats to determine which side an odor arrives [11] and a rat’s ability to follow a scent trail is degraded when a single nostril is blocked [12]. Stereo-olfaction has also been shown to play a role in odor localization and tracking in moles [10] and humans [13].

Most previous comparative studies on animal olfactory capabilities have focused on measures reflecting olfactory sensitivity. For example, neuroanatomy and skull morphology have been shown to be strongly correlated with olfactory receptor gene repertoires in mammals, thus serving as a viable metric for olfactory capacity across species [14]. Conditioning paradigms and behavioural assays have been used to evaluate sensitivity to different chemical compounds in some mammals, mainly in mice [15,16] and primates [17–19]. However, measures of olfactory sensitivity and discrimination cannot tell us very much about the behavior animals use to track an odor to its source. External nasal morphology may give insight into what behavioral mechanisms animals are using to locate odor sources, particularly anterior nare placement and spatial olfactory information. In this study, we used phylogenetic comparative methods to test the hypothesis that external nasal morphology should vary with potential olfactory tracking capabilities.

**Fig 1:**
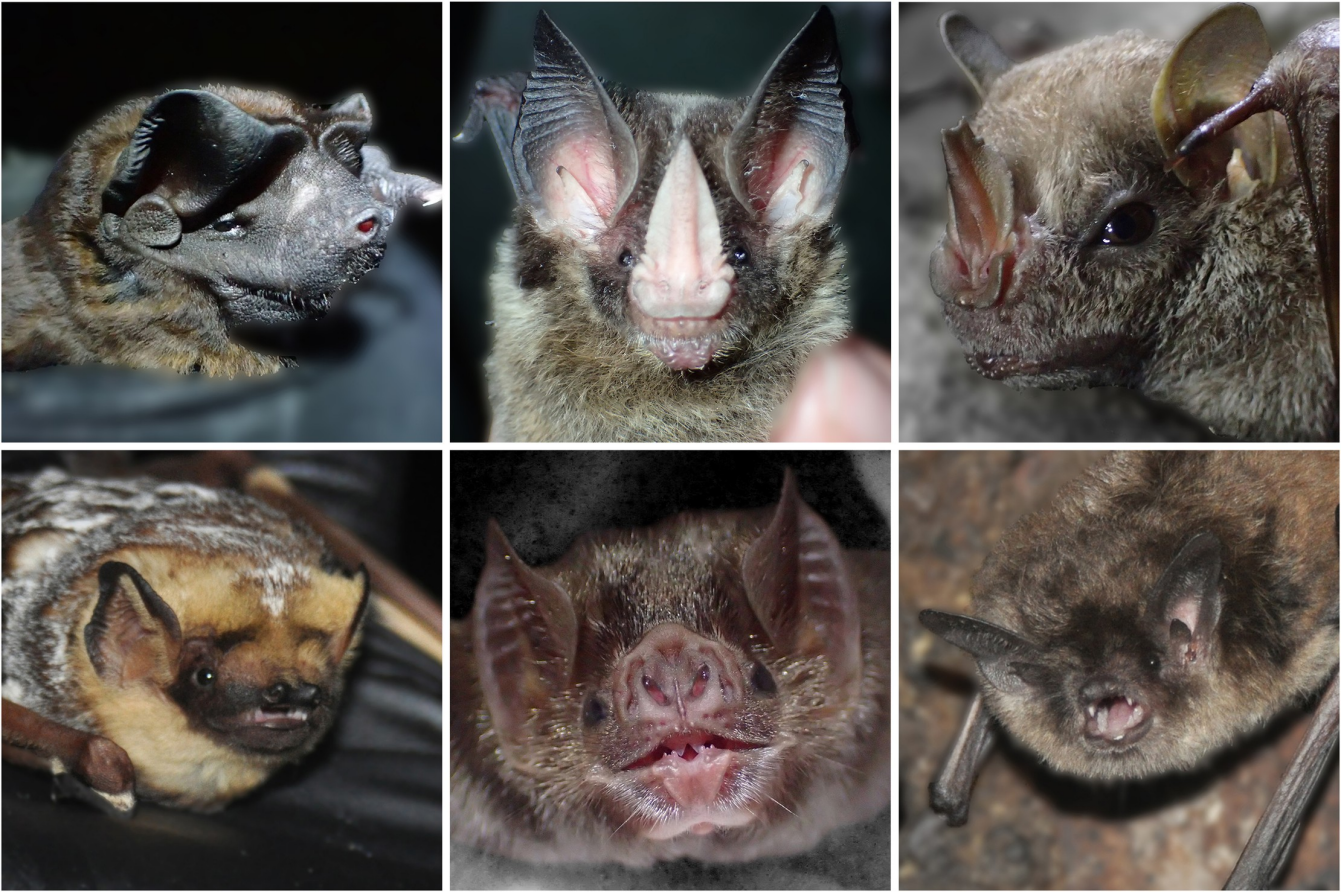
Examples of nose shape, nostril shape, and nostril positioning of species included in this study. *Top*, *left to right*: Black mastiff bat (*Molossus rufus*), Striped hairy-nosed bat (*Gardnerycteris (Mimon) crenulatum*), Hairy big-eyed bat (*Chiroderma villosum*). *Bottom*, *left to right*: Hoary bat (*Lasiurus cinereus*), Common vampire bat (*Desmodus rotundus*), Yuma myotis (*Myotis yumanensis*). Photographs by A.F. Brokaw.

With over 1,400 species and considerable variation in morphology and ecology, bats offer many opportunities for investigating ecological and evolutionary questions in a comparative framework. Olfaction is used during foraging by many bat species, particularly fruit and nectar feeding bats [20–24]. Seba’s short-tailed fruit bats (*Carollia perspicillata*) display enhanced sensitivity to fruit-typical odor compounds and can discriminate odor quality and quantities, a first step in being able to recognize and follow a concentration gradient [25,26]. Frugivorous bat species have enhanced olfactory acuity and increased reliance on olfactory cues [27,28]. Bats are hypothesized to use olfactory cues for initial detection and discrimination at long distances, followed by echolocation for exact localization at close distances [22]. In environments with high background clutter (such as forest understory), echolocation may be an inefficient mechanism for detecting objects even at close ranges, making olfactory cues all the more important for detecting and localizing food resources [29]. Bats also display a surprising diversity of nostril size, shapes, and orientation (Fig 1), the drivers of which are still not well understood (but see [30]).

In this study we evaluate if there is a relationship between external nasal morphology and foraging ecology among bats. As flying vertebrates, bats face a more complex fluid environment for olfactory tracking than terrestrial animals, while also having less behavioral flexibility to compensate for these challenges (such as ability to slow down or pause while sampling). Stoddart [31] proposed that wider separation between receptors (e.g. external nares in vertebrate) may enhance olfactory tracking navigation by increasing the effective sampling area of an organism or by increasing the ability to detect and resolve differences in odor concentration [3,32] or arrival timing [4]. Based on these hypotheses, we predicted that bat species known to use odor while foraging would have broader separation of the nostrils compared to species that predominantly rely upon acoustic cues (i.e. insectivorous bats). Foraging habitat and flight capabilities may also exert selective pressure on olfactory tracking and nasal morphology, by changing the relative importance of sensory inputs (i.e. odor and echolocation in cluttered habitat [29]), or ability to move or change speed within the odor plume (i.e. flight manoeuvrability). Bats that forage in open environments or that have limited manoeuvrability while foraging (high aspect ratios, fast flight speeds) would be more constrained by their sampling ability, and would be predicted to have wider nostrils than more manoeuvrable species.

## Materials and methods

### Morphological data

**Fig 2:**
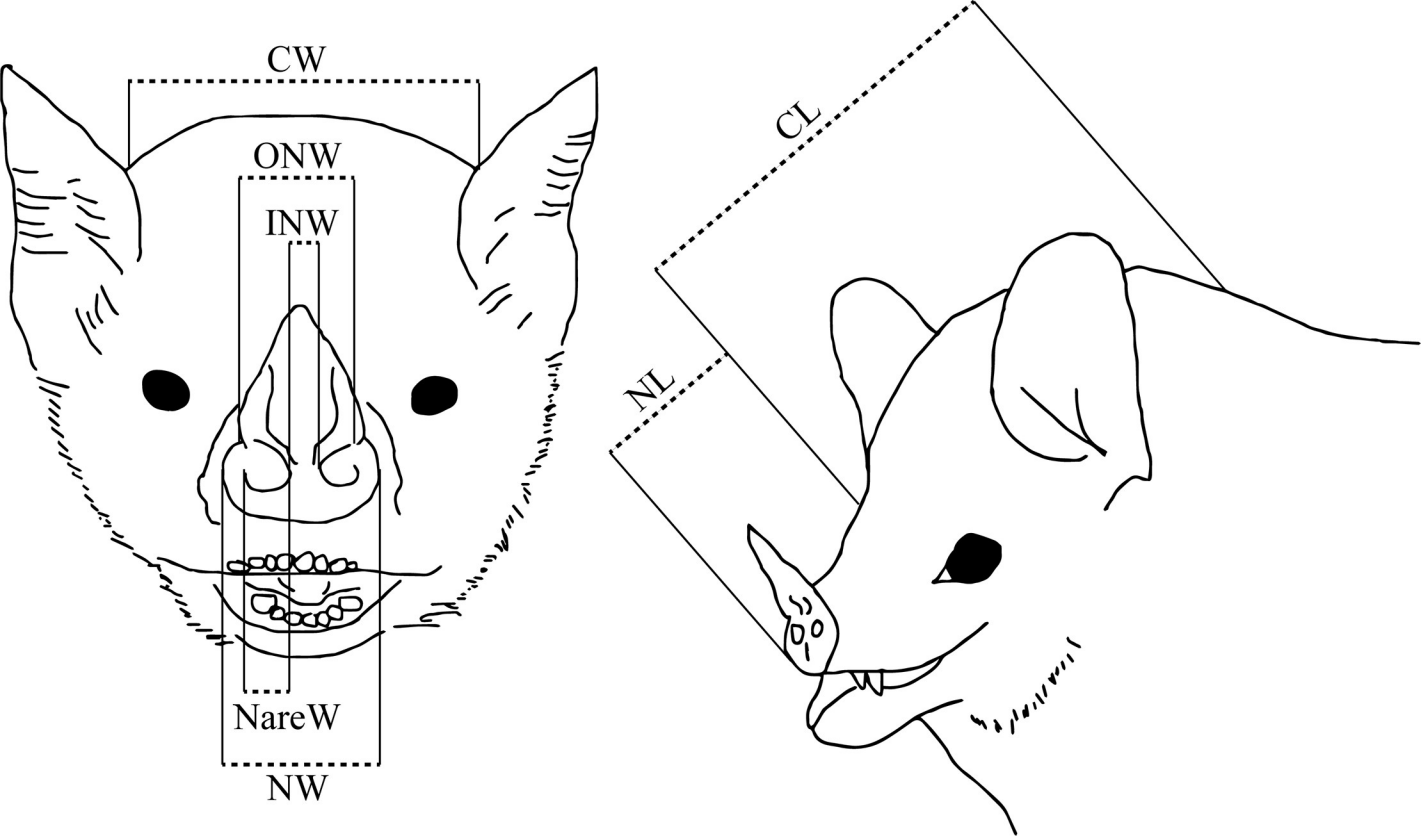
Facial morphological characters examined from alcohol-preserved museum specimens. See Table 1 for abbreviations and descriptions.

We measured eight external nasal and body measurements on 40 New World bat species from four different families (Molossidae, Mormoopidae, Phyllostomidae and Vespertilionidae) (Fig 2, Table 1). Measurements were taken from a total of 328 alcohol-preserved specimens, located at the Biodiversity Teach and Research Collection at Texas A&M University (College Station, Texas, USA). We recorded measurements from between two and 11 individuals per species (with an average of eight individuals measured per species) (S1 Table). All linear external measurements were taken to the nearest 0.01 millimeter using digital calipers. All forearm measurements were taken from the right wing, where possible. The inner nostril width ratio (INWR) was calculated by dividing the cranial width by the inner nostril width, after Stoddart [31]. To reduce measurement error, all samples were measured by the same person (A.F.B.). For each species, measurements from each character were examined for outliers and these specimens were excluded from analysis to further reduce measurement bias. Only specimens that were intact with no bone or organ removal, and soft tissues (especially nose-leaves) in a natural position with no severe angles or deformation were selected for measurements. Alcohol and other preservation methods for animal specimens can have strong effects on measurements such as body mass [33], and metadata for alcohol-preserved specimens are rarely consistently recorded or digitized. Therefore, average body mass for each species was obtained from the PanTHERIA database [34], with the exception of *Molossus rufus* [35], *Myotis nigricans* [36] and *Lonchophylla handleyi* [37].

**Table 1:** Description of morphological characters.

Character	Description
**INW**	*inner nostril width*: minimum distance between the inner edges of the external nares
**ONW**	*outer nostril width*: minimum distance between the outer edges of the external nares
**NL**	*nose length*: distance from tip of nose to midpoint between the eyes
**NW**	*nose width*: maximum distance between the outer part of the nose/rhinarium
**CL**	*cranial length*: distance from base of occipital bone to midpoint between the eyes
**CW**	*cranial width*: maximum distance of the head measured immediately anterior to the ears
**INWR**	*inner nostril width ratio*: ratio of the cranial width to the inner nostril width
**NareW**	*nare width*: average maximum distance across the external nares

All characters were measured in millimeters (mm).

To try to estimate potential shrinkage effects of preserved specimens, we collected morphometric data from live bats (n = 4–6 each) for 10 species included in this analysis. Live bats were captured using mist-nets in Lamanai, Orange Walk District, Belize (17.75117 N, −88.65446 W) and were released following processing. All methods were approved by the Belize Forest Department (permit number FD/WL/1/19(10) to A. Brokaw) and the Texas A&M University Institutional Animal Care and Use Committee (AUP 2017–0139). We calculated the percent change between live animals and preserved specimens for each morphological character (Fig 2, Table 1), for each species. Specimens had an average loss of 2.67% across all morphological characters. Forearm had a lower average percent shrinkage than all other measurements (mean = -1.76%, one-way ANOVA, *F =* 3.284, *P =* 0.007) (S1 File). There was no difference in percent shrinkage across morphological variables when compared between species (one-way ANOVA, *F =* 0.504, *P =* 0.868). While specimens did show shrinkage compared to live animals, the difference was consistent across measurements and species, so we feel confident that any differences observed in our dataset reflect the variation in living organisms.

Previous studies on olfactory search strategies in mammals have shown the importance of stereo-olfaction and tropotaxis in rats [11,12] and mice [38]. While not included in the statistical analyses, we also collected morphometric data from alcohol-preserved museum specimens of rats (*Rattus rattus*) and mouse (*Mus musculus*) as a reference to compare to bat values.

### Ecological data

Using published data, we classified each species into categories that reflect their foraging and flight behavior (S1 Table). Species were assigned to one of five dietary categories. Bats whose diets are known to contain large proportions of both plant and animal material were classified as omnivores. Foraging habitat and mode were assigned from the literature, modified from the classification scheme presented by Denzinger and Schnitzler [39] to serve as a proxy for overall flight abilities. Habitat and vegetation complexity can have an effect on the distribution of odors in the environment, thus influencing movement of odor plumes [40]. We define three types of foraging habitat, based on the amount of environmental clutter: open space, edge space and narrow space. Foraging mode refers to method of prey acquisition: aerial (acquire prey from air) or gleaning (taking food from off a surface). For a subset of species, we also recorded average flight speed (21 species), wing loading (25 species) and aspect ratio (35 species) from the existing literature (S1 Table), to quantitatively evaluate the relationship between flight ability and nose morphology. External nasal morphology is also mechanically linked to echolocation in species that emit echolocation pulses through their nostrils [41], so each species was classified as either a nasal or oral emitting echolocator. Bats were classified as either migratory (undergoing long-distance seasonal migration) or non-migratory, based on existing literature.

### Statistical analyses

**Fig 3:**
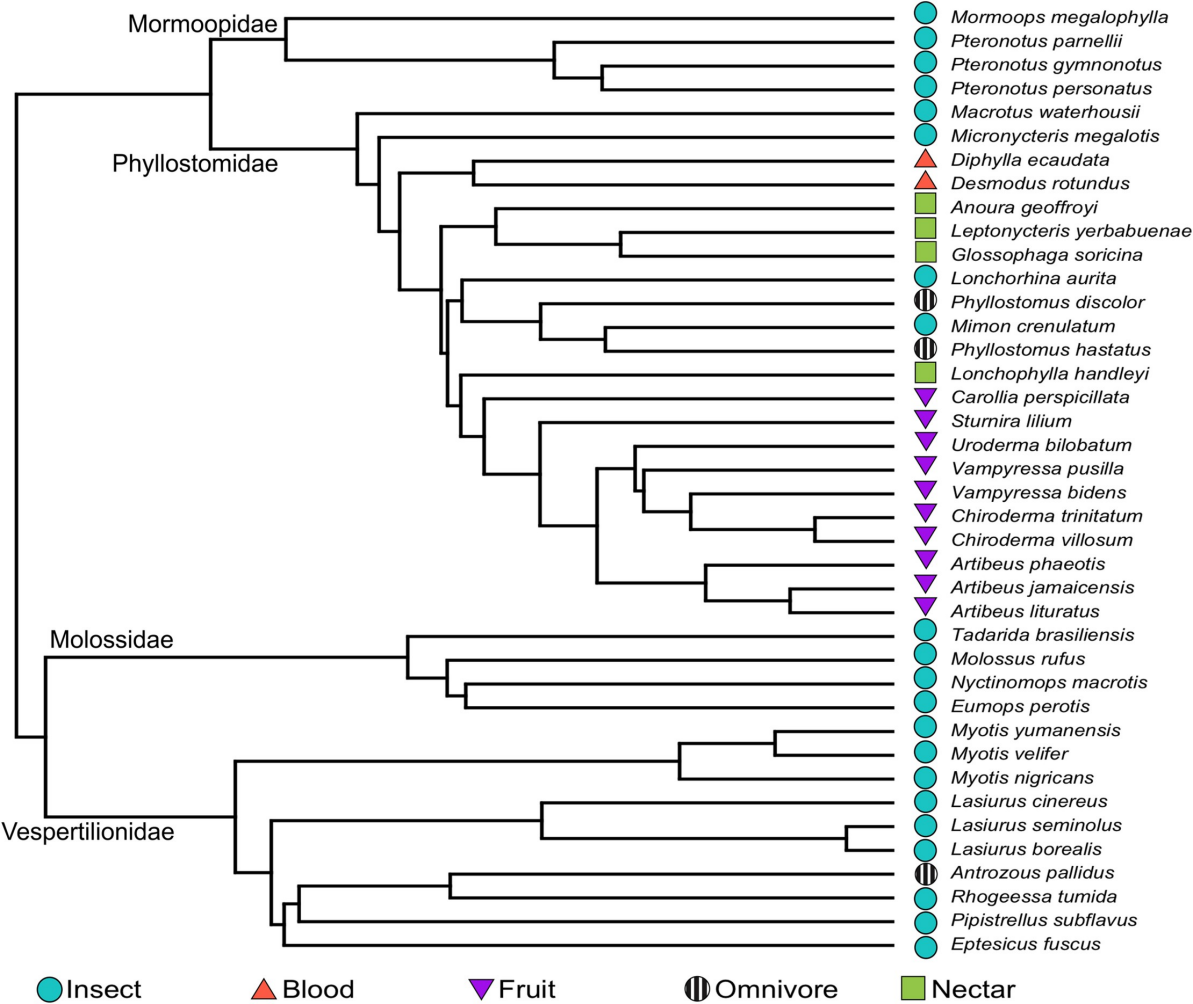
Phylogenetic tree used for phylogenetic comparative methods. Tree is derived from the species-level phylogeny of Shi and Rabosky [43] and pruned to include only species examined in this study. Symbols represent diet categories.

Closely related species tend to resemble one another, resulting in lack of statistical independence and pseudo-replication [42]. To account for shared ancestry, we performed all analyses within a phylogenetic context. We based our phylogeny on the one used by Shi and Rabosky [43], a time-calibrated, maximum-likelihood phylogeny of extant bats based on a 29-locus genetic supermatrix, which we then pruned to include only the 40 species in this study (Fig 3). We performed all analyses using the ‘ape, ‘caper’, ‘geiger’ and ‘phytools’ packages in R, version 3.5.0 [44–47]

Prior to analysis, all morphological variables were log-transformed to meet the assumptions of normality and the data was inspected for outliers. The phylogenetic signal for morphological variables were estimated using Pagel’s λ and Blomberg’s *K* (*phylosig* function, *phytools*;[47]). Pagel’s λ [48] is an estimate of the correlation between species relative to the correlation expected under Brownian evolution, on a scale between 0 (no correlation between species, equal to a star phylogeny) and 1 (correlation between species equal to Brownian expectation). Blomberg’s *K* is a measure of the partitioning of variance among clades. *K* < 1 indicates that closely related species are less similar to each other than expected by Brownian motion, while *K* > 1 means that closely related species are more similar to each other than expected [49].

We were interested in how morphology of the nose and head, particularly width of the nostrils, is influenced by diet and other ecological variables. However, nearly all measured morphological variables were highly correlated with each other, even when accounting for phylogenetic non-independence (PGLS regressions, *P < 0*.*05*). We performed a phylogenetically informed principal component analysis (pPCA) on the mean morphological variables to explore the co-variation between variables and obtain independent axes of variation, using *phytools* [47]. Cranial width and inner nostril width were excluded from the pPCA because they were used to calculate the inner nostril width ratio. Relationships between the head and nose morphology and ecological variables were tested using phylogenetic generalized least squares (PGLS) regressions based on species’ scores obtained from the pPCA (*pgls* function in the *caper* package [45]). The optimal value of lambda was estimated using maximum likelihood during calculation of the PGLS. We regressed each principal component (PC) separately on the ecological variables. To control for differences in body size across species, we used body mass and average forearm length as covariates in the models, modelled separately. Size measures were used as covariates instead of size-corrected residuals because the morphological variables were collinear, and the use of residuals in model fitting can result in biases in phylogenetic data [50]. Model selection was performed through comparisons using Akaike Information Criteria corrected for small samples sizes (AICc, [51]).

Principal component values can be difficult to interpret in a biological context, so we also compared the inner nostril width relative to head (INWR) of bats across different ecologies. This is the measure most directly related to separation of air streams between the two nares, and is therefore hypothesized to be the most relevant to potential olfactory tracking mechanisms, such as tropotaxis [31]. We used phylogenetic ANOVAS to test for differences across diet, foraging habitat, and echolocation mode, and conducted *post-hoc* comparisons of means for the statistically significant tests, adjusted using the Holm-Bonferroni correction to account for multiple testing.

Roughly half of the species in this study belong to one family (Phyllostomidae, 22 species), all of whom are primarily nasal echolocators. Phyllostomidae is a highly diverse group of bat species with a wide range of ecological variation in morphology, diet and ecology [52]. We applied the above analyses just within this family, to see if and how these relationships change across and within the phylogeny.

### Data accessibility

We provide museum catalogue numbers and raw measurements as a supplement in S1 Appendix.

## Results

### Phylogenetic signal

**Fig 4:**
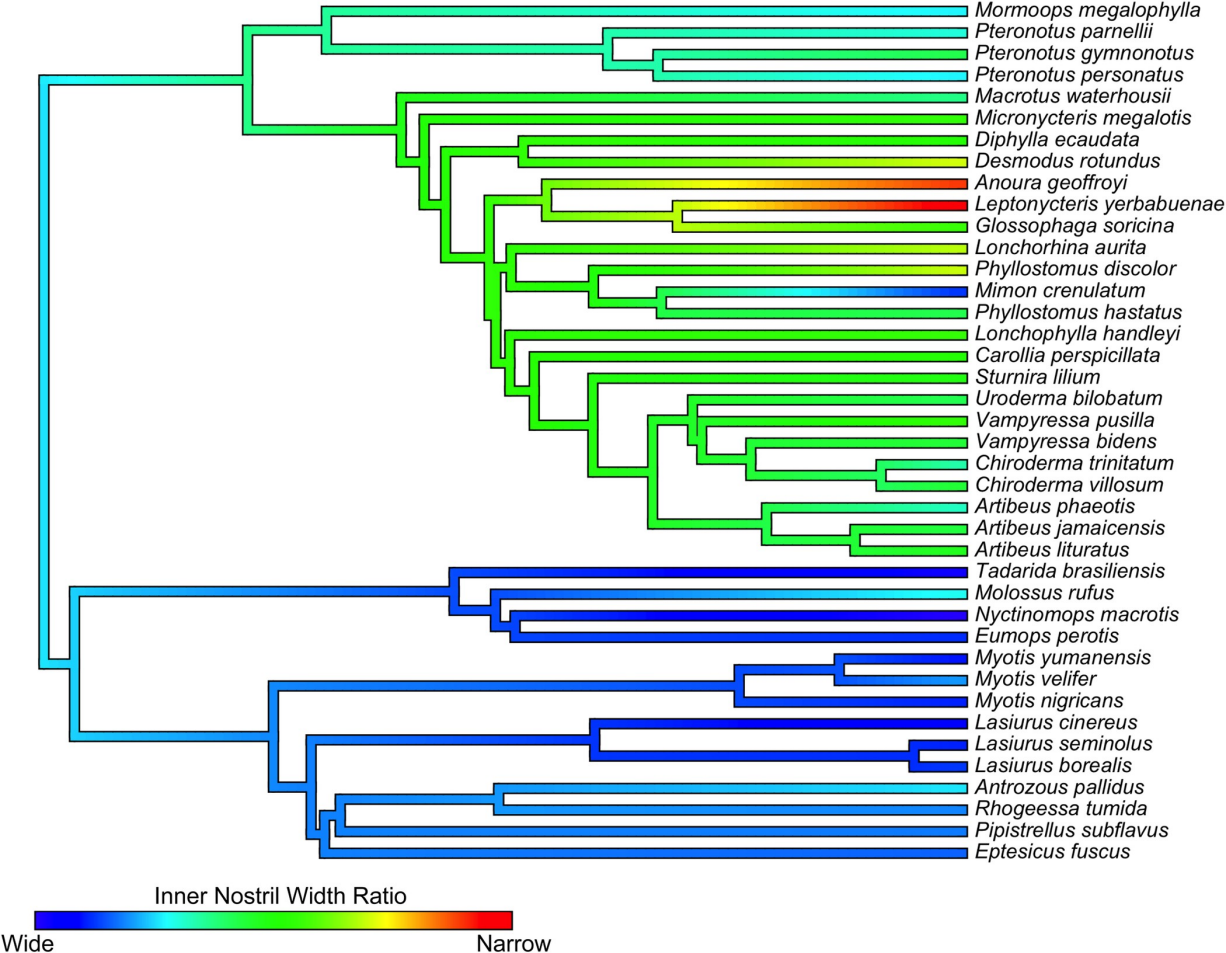
Ancestral character estimation of inner nostril width ratio (INWR). Red colors indicate species with narrow nostrils relative to head width, while dark blue indicates species with relatively wide nostrils. Illustration made in R (*contMap* function, package *phytools;*[47]).

We found weak signal (less than expected under Brownian motion) for both average mass and forearm length (Blomberg’s K < 0.6, P > 0.05; Pagel’s λ < 0.5, P > 0.05). Cranial width also showed a weak phylogenetic signal, which was further reduced when *Eumops perotis* was excluded from the analysis. Measurements of nose morphology (INW, ONW, NL, NW, and INWR) showed strong phylogenetic signal (Blomberg’s K > 0.7, P < 0.05; Pagel’s λ > 0.8, P < 0.05), implying that neighboring species have more similar nose morphology than expected under Brownian motion of evolution (Fig 4). When looking only at the Phyllostomidae, there was very weak phylogenetic signal for nearly all morphology variables measured in this study, further suggesting a non-random pattern in morphology across the entire dataset (S2 File).

### Variation in nose and cranial morphology

Morphometric analysis reveal substantial variation in the nose and head morphology among bats. A summary of morphological measurements for species included in this study is given in Table 2. Linear inner nostril separation distances from ranged from less than 1 mm (0.77 mm in *Anoura geoffroyi*) to 5.6 mm in *Eumops perotis*, compared to an average 2.01 mm from rodent specimens. The greatest relative separation (INWR) in bats was found in *Nyctinomops macrotis* (3.2) and smallest relative separation in the nectar-feeding species *Leptonycteris yerbabuenae* (14.88).

**Table 2:** Summary of morphological measurements for species included in this study.

Taxon	INW	ONW	NL	NW	CL	CW	NareW	INWR	FA
***Eumops perotis***	5.59	9.05	19.04	10.54	23.61	25.32	1.73	4.53	76.35
***Molossus rufus***	2.47	4.11	7.55	5.68	16.52	16.36	0.82	6.74	49.73
***Nyctinomops macrotis***	4.39	5.85	8.21	7.10	16.04	14.01	0.73	3.20	61.85
***Tadarida brasiliensis***	2.92	4.14	7.91	5.29	13.04	10.12	0.61	3.47	42.91
***Mormoops megalophylla***	1.69	3.01	5.26	4.02	9.97	10.79	0.66	6.42	54.56
***Pteronotus gymnonotus***	1.22	2.79	6.80	4.09	11.72	10.00	0.79	8.30	52.79
***Pteronotus parnellii***	1.86	3.47	9.04	4.52	13.79	12.56	0.81	6.85	59.35
***Pteronotus personatus***	1.28	2.53	5.14	3.45	10.70	8.07	0.62	6.32	43.08
***Anoura geoffroyi***	0.77	2.10	8.29	4.51	15.75	11.04	0.67	14.66	42.46
***Artibeus jamaicensis***	1.79	4.49	7.78	7.49	19.51	15.17	1.35	8.48	59.59
***Artibeus lituratus***	1.85	4.86	7.18	7.83	19.39	15.90	1.51	8.71	64.65
***Artibeus phaeotis***	1.43	3.23	5.54	6.25	14.10	9.92	0.90	7.06	38.32
***Carollia perspicillata***	1.15	2.95	5.68	5.40	15.41	11.16	0.90	9.81	44.09
***Chiroderma trinitatum***	1.41	3.27	6.06	6.44	14.98	10.77	0.93	7.72	38.76
***Chiroderma villosum***	1.56	3.65	7.29	8.28	17.98	13.17	1.04	8.59	45.83
***Desmodus rotundus***	1.17	3.34	4.94	7.83	18.49	13.85	1.09	11.97	59.72
***Diphylla ecaudata***	1.35	3.53	3.07	5.68	19.08	12.86	1.09	9.56	54.83
***Glossophaga soricina***	1.08	2.75	5.66	4.21	14.27	10.56	0.83	9.91	35.92
***Leptonycteris yerbabuenae***	0.87	2.64	9.76	4.75	18.22	12.80	0.89	14.88	53.43
***Lonchorhina aurita***	1.04	3.09	7.39	4.86	15.43	11.76	1.02	11.45	45.75
***Lonchophylla handleyi***	1.19	3.23	10.82	4.77	17.18	12.05	1.02	10.15	44.92
***Macrotus waterhousii***	1.50	3.50	5.59	5.16	13.55	11.45	1.00	7.65	53.49
***Mimon crenulatum***	2.50	4.08	6.64	7.19	14.50	11.54	0.79	4.63	48.92
***Micronycteris megalotis***	0.94	2.36	6.11	5.02	11.29	9.15	0.71	9.80	35.65
***Phyllostomus discolor***	1.31	3.83	10.09	7.32	19.94	15.56	1.26	12.10	62.87
***Phyllostomus hastatus***	2.12	5.58	10.65	9.64	21.85	17.00	1.73	8.09	73.82
***Sturnira lilium***	1.29	3.32	4.95	4.81	15.23	12.29	1.02	9.53	38.92
***Uroderma bilobatum***	1.43	3.59	6.21	5.78	15.44	11.09	1.08	7.87	44.62
***Vampyressa bidens***	1.10	3.03	5.59	6.52	13.57	9.11	0.96	8.33	35.85
***Vampyressa pusilla***	0.91	2.15	4.39	4.65	11.75	8.92	0.62	9.81	31.63
***Antrozous pallidus***	1.97	3.29	5.96	5.58	13.67	12.49	0.66	6.38	50.78
***Eptescius fuscus***	2.30	3.88	7.44	4.66	12.54	11.62	0.79	5.11	48.89
***Lasiurus borealis***	2.11	3.32	4.15	4.19	9.38	9.52	0.61	4.53	40.12
***Lasiurus cinereus***	3.09	4.85	5.96	5.83	13.09	11.35	0.88	3.67	52.35
***Lasiurus seminolus***	2.14	3.34	3.88	4.14	10.16	9.55	0.60	4.47	40.00
***Myotis nigricans***	2.42	3.91	7.11	4.81	11.35	10.64	0.75	4.44	35.91
***Myotis velifer***	1.79	3.16	5.93	3.97	11.03	10.12	0.69	5.74	43.86
***Myotis yumanensis***	2.02	2.72	6.21	3.05	9.51	8.62	0.35	4.28	35.12
***Perimyotis subflavus***	1.45	2.30	4.94	2.82	8.16	8.40	0.43	5.84	33.53
***Rhogeessa tumida***	1.65	2.88	5.58	3.58	9.49	8.87	0.62	5.45	30.70
**Mean**	1.80	3.58	6.89	5.54	14.52	11.87	0.89	7.66	47.65
**SE**	0.15	0.19	0.42	0.27	0.58	0.49	0.05	0.46	1.76
***Rattus rattus***	2.52	4.24	20.09	4.93	27.06	16.28	0.86	6.63	-
***Mus musculus***	1.50	2.39	9.65	3.10	16.28	10.63	0.44	7.43	-
**Mean**	2.01	3.31	14.87	4.01	21.67	13.46	0.65	7.03	-
**SE**	0.51	0.93	5.22	0.91	5.39	2.82	0.21	0.40	-

Morphology character abbreviations are described in Table 1. All measurements except INWR are in millimeters.

A phylogenetic principal component analysis (pPCA) on the mean morphological variables yielded three component axes that jointly explain 93.8% of the variation (S3 File). The first component (PC1, 67.3% of variance) was strongly affected by ONW (loading = -0.911), NW (-0.897), CL (-0.906), and NareW (-0.915), indicating an overall measure of face and nose size. *E*. *perotis* and *Phyllostomus hastatus* scored high on this axis, indicating large and wide noses. The second axis (PC2, 15.5%) had a strong, positive loading on INWR (+0.906), and separated species with nostrils wide relative to head width (e.g. *N*. *macrotis* and *Tadarida brasiliensis*) from species with narrow set nostrils (e.g. *L*. *yerbabuenae* and *A*. *geoffroyi*). A third axis (PC3, 10.9%) was most strongly affected by NL (+0.587), separating species based on nose length.

**Fig 5:**
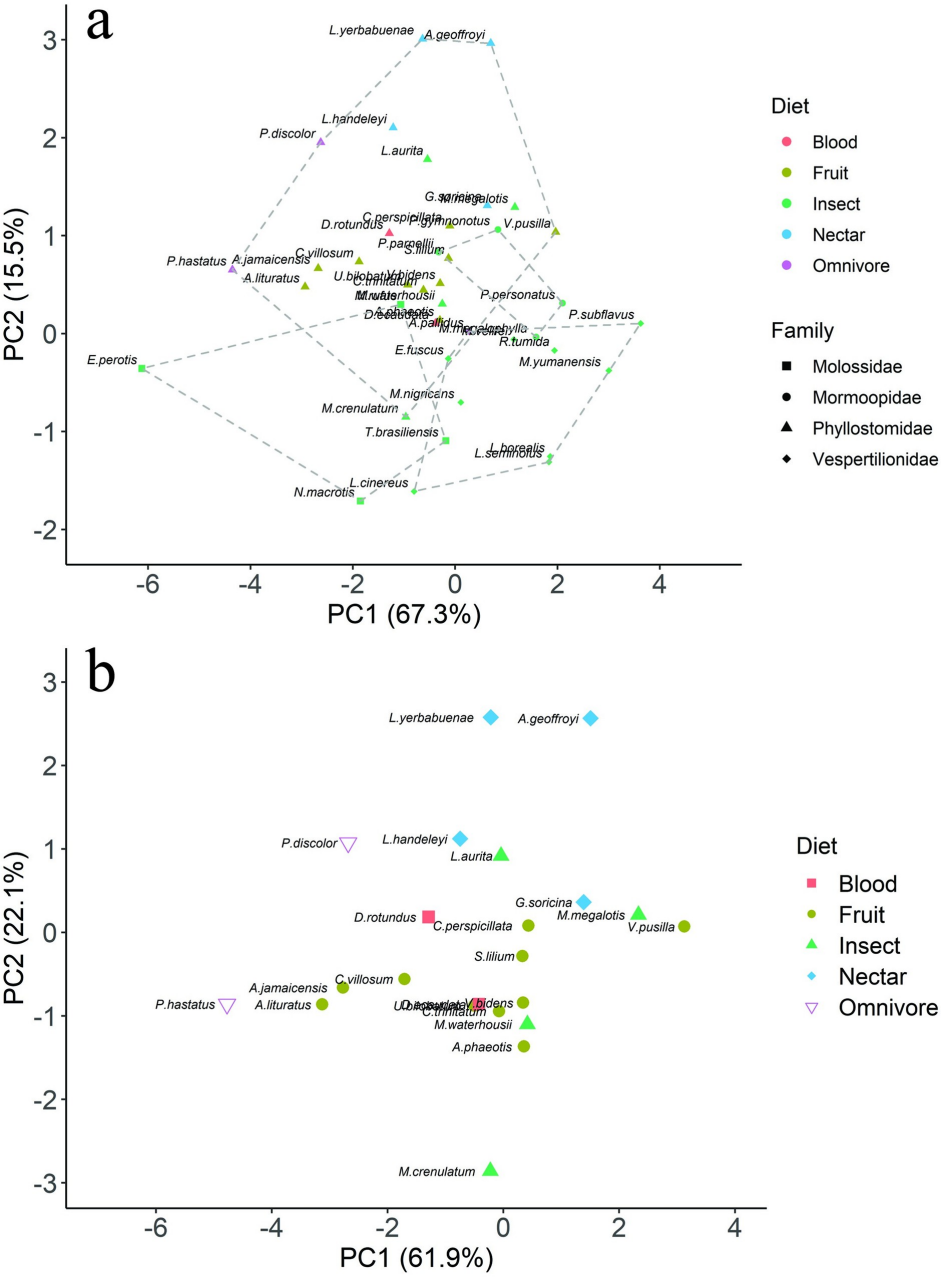
Ordinations of the first and second components of the phylogenetic principal component analysis (pPCA). pPCA results for the full dataset (*a*) and just Phyllostomidae (*b*). Dotted lines for the full dataset delineate minimum convex hulls for each family.

There was no particular pattern across clades or diet along the first axis (Fig 5A), though the insectivorous species tended to be smaller (positive loading), with fewer large bat species. Bats in the family Phyllostomidae tended to have high, positive loadings on the PC2, indicating narrow nostrils relative to head size. Across families, insectivores tended to have negative loadings on this second axis, suggesting relatively wider nostrils.

A pPCA using only species in the family Phyllostomidae yielded similar patterns, with three component axes jointly explaining 91.9% of the variance. The first PC (61.9% of variance) was strongly affected by ONW (-0.895), NW (-0.828), CL (-0.922) and NareW (-0.939), with larger species scoring high on this axis (e.g. *Phyllostomus hastatus*). The second axis (PC2, 22.1%) also had a high, positive loading on INWR (+0.947), separating species with narrow set nostrils from species with wider nostrils. Most species fell intermediate to extremes on both axes, with no obvious pattern across different diets (Fig 5B).

### Relationship between morphology and ecology

**Fig 6:**
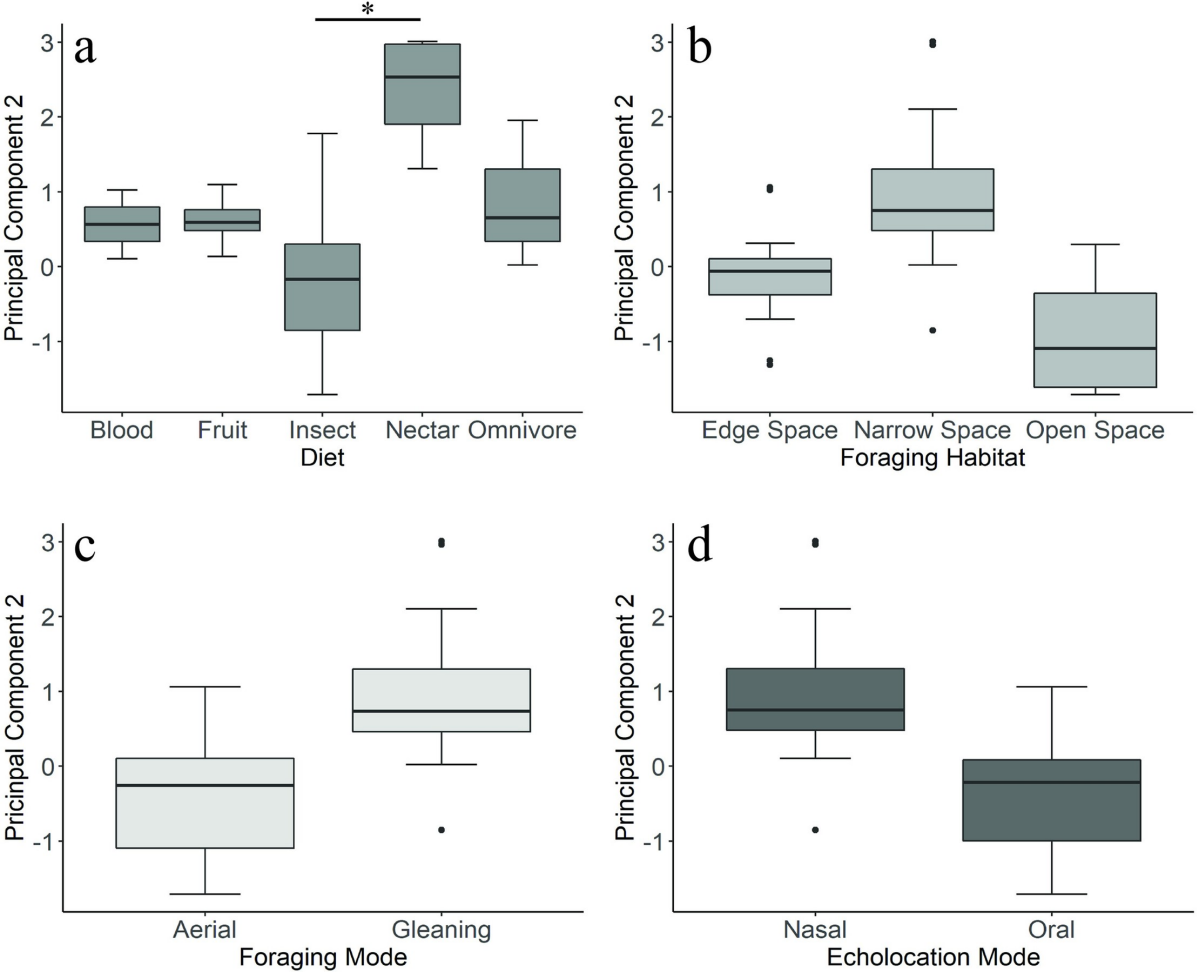
Boxplots representing variation in PC2. Data from the full dataset for four of the measured ecological variables: diet (*a*), foraging habitat (*b*), foraging mode (*c*), and echolocation mode (*d*). Only diet had a significant effect on PC2 in a PGLS regression. Asterisks indicate statistical significance in a phylogenetic ANCOVA.

Using scores computed on the pPCA axes for each species, we constructed models to investigate the relationship between morphology and ecology. We ran multiple regressions with each principal component as a dependent variable, using either body mass or forearm as a covariate. For simplicity, we only present the results for regressions using body mass as the covariate, though regressions run using forearm as a size covariate produced comparable results (S4 File). For PC1, models with the strongest statistical support included the independent influences of foraging habitat, echolocation mode and migratory behavior. The least complex models with higher statistical support (ΔAICc < 2) included foraging habitat, echolocation mode, and migration pattern. Size was a significant predictor for all models (*P* < *0*.*01*). Foraging habitat also had a significant effect on the response variable (*P < 0*.*05*), where species that forage in more open habitats tended to have larger head and body sizes (S1 Fig). For PC2, the most relevant predictors were diet, foraging habitat, and echolocation mode. Diet was a significant predictor across all of the models with the most statistical support (*P <* 0.001). Nectar feeding bats had significantly higher loadings on PC2 compared to insectivores (*t* = 5.73, *P =* 0.02) (Fig 6).

**Fig 7:**
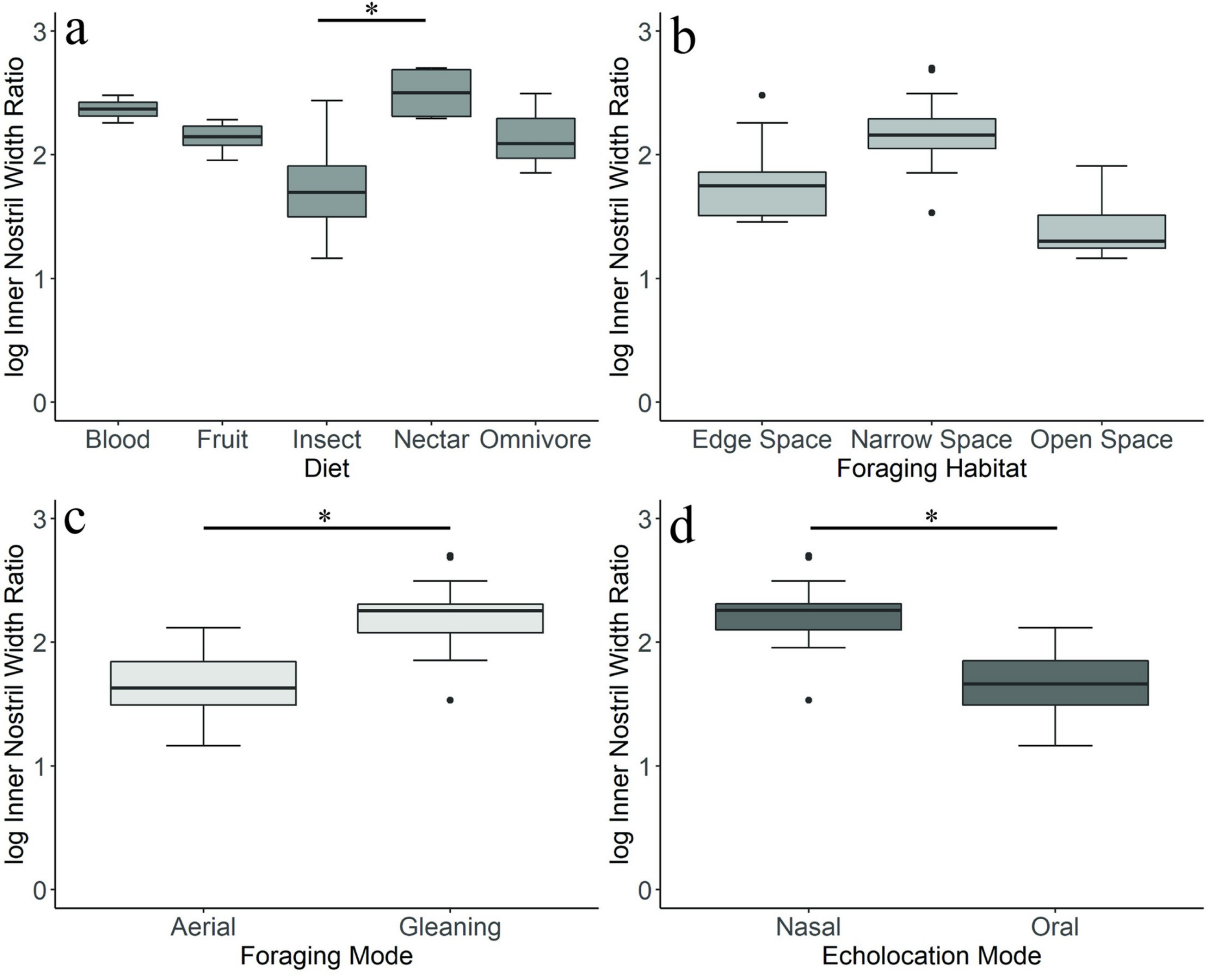
Boxplots representing variation in inner nostril width ratio (INWR). Data from the full dataset for four of the measured ecological variables: diet (*a*), foraging habitat (*b*), foraging mode (*c*), and echolocation mode (*d*). Asterisks indicate statistical significance in a phylogenetic ANOVA.

Mean relative nostril width (INWR) differed significantly across bats with different diets (phylogenetic ANOVA, *F =* 10.459, *P =* 0.018). Bats that feed primarily on nectar had narrower nostrils compared to insect-eating bats (Holm-Bonferroni adjusted, *t =* -5.19, *P =* 0.01, Fig 7A). Bats that forage via gleaning also had narrower nostrils compared to aerial foragers (*F =* 49.49, *P =* 0.018, Fig 7C), as did bats that echolocate nasally (*F =* 52.605, *P =* 0.017, Fig 7D).

When comparing only between species in the family Phyllostomidae, diet was still a significant predictor for PC2 axis (*P =* 0.026), but inner nostril width ratios were not significantly different across diets (*F =* 4.39, *P =* 0.123) (S5 File). Using PCs and PGLS, we also tested for a relationship between morphology and quantitative flight characteristics (average speed, wing loading and aspect ratio). There was no significant covariation between the scores of either principal component axis and any of the flight traits (*p* > 0.05) (S6 File).

## Discussion

Greater separation of the airstreams passing over the olfactory receptors is hypothesized to facilitate simultaneous comparison of the olfactory environment on opposite sides of the face, useful for olfactory tracking via tropotaxis. This may be especially true in organisms with limited post-cranial mobility or those moving at fast speeds in three-dimensional environments [4,31,32]. Using bats as a model, we evaluated if there is a link between external nasal morphology and potential olfactory tracking behavior. Contrary to our predictions, bat species that rely on olfaction for foraging had narrower nostrils compared to species that rely primarily on echolocation or hearing for foraging, even when controlling for evolutionary history. It remains unclear what, if any, ecological factors may be driving the diversity of bat nasal morphology.

Phylogenetic history had a significant influence on the external morphological characters measured in this study, indicating that closely related species are more likely to resemble each other than distantly related species. However, this signal was reduced or non-existent when comparing external nasal morphology within the family Phyllostomidae, consistent with previous studies investigating the role of ecological factors in driving morphological diversity in the family [52–54].

Contrary to our predictions, insectivorous bats across all families had wider nostrils (lower INWR), while nectar feeding species had the narrowest nostrils. This pattern was also detected even within just the family Phyllostomidae, as insect-eating species in our dataset trended towards wider nostril separation (though with a lot of variation). This is surprising given the well-documented use of olfaction by fruit and nectar-eating bats during foraging. In many species of plant-visiting phyllostomid bats, appropriate odor cues are necessary to stimulate foraging, even in the absence of other food-related cues (such as shape or texture) [21,22,55]. Bats are attracted to odor lures in the field, with more captures recorded in odor-baited mist nets [20,56] and increased frugivore activity around fruit odor lures in open field areas [57]. Olfactory cues may play an role in the detection of ripe fruits or flowers over long distances [22,58], as well as facilitating foraging in cluttered habitats [29]. However, it may be that bats rely more on spatial memory to locate potential food resources [59] and then rely on olfactory cues for fine-scale localization and discrimination.

Tropotaxis is not the only behavioral strategy animals might use to follow olfactory stimuli, and it may be that fruit and nectar feeding bats rely on other strategies to follow odor plumes while foraging. Increased manoeuvrability could compensate for narrow nostrils by allowing bats to quickly sample an area via klinotactic (serial sampling) mechanisms. Frugivorous and nectarivorous species are generally well adapted for foraging in clutter habitats, with short, broad wings that allow for hovering and manoeuvring around obstacles [60,61]. Habitat also has an effect on the structure of odor plumes. Wind speeds tend to be lower in forested areas, creating longer bursts of odor signals in the air over father distances [40]. However, vegetation can also cause continual shifts in wind and odor direction, making plume following more difficult [62]. Under these conditions, behavioral rather than morphological adaptations would be needed to support odor tracking.

Morphological adaptations for odor tracking may be constrained by selection for other purposes. Multiple studies in Phyllostomid bats have shown that adaptive shifts in cranial size, shape, and bite force are strongly associated with feeding mechanics [53,63,64]. In frugivorous species, this is frequently characterized by shorter rostra and mandibles, and more robust crania [52,53], while nectarivorous bats instead display elongated rostrums, thought to be associated with the development of an elongated tongue [53,63,64]. While cranial and external soft tissue morphology are likely correlated, the extent to which these units might evolve together is unknown. Thus, it is possible that while natural selection acted on cranial morphology associated with the manipulation and processing of food, there was less selective pressure on external characteristics, leading to a mismatch in features resulting in a larger heads or longer noses while keeping external characteristics such as nose shape the same.

Feeding ecology is unlikely to be the only driver of this morphological variation in external nose morphology. Within our dataset, diet only explained about 36% of the variation in external nasal morphology, and foraging habitat explained only 11% of total variation. All of the fruit and nectar feeding species in this dataset are found within the family Phyllostomidae, a group of nasal-emitting echolocators. Nasal emitting bats were shown to have significantly narrower nostrils relative to head size than oral emitting bats. This presents an interesting potential trade-off between sound emission and olfactory tracking via tropotaxis in nasal emitting bats. While nostril separation in long duration constant-frequency emitting bats (Rhinolophidae and Hipposideridae) is tightly linked to echolocation parameters [65], it is unknown if the short broadband, frequency modulated calls of Phyllostomid bats are similarly influenced by nose morphology. Future work comparing these distinct groups could help disentangle the selective pressures of nasal echolocation on external nose morphology. Bats in the family Pteropodidae are also known to rely on olfactory cues while foraging [23,66–68], but do not echolocate laryngeally. It might be interesting to further test the relationship between foraging ecology and external nasal morphology within those species, without the confounding effects of echolocation.

External nare shape and orientation likely play an important role in the control of airflow into the nasal cavity, for both respiration and olfaction [69–71]. During sniffing in dogs, air is inhaled from the front and exhaled to the side, which alters airflow rates and permits more efficient sampling of odorants [72]. Interestingly, bats appear to be different from rodents and dogs in that some air may pass through the olfactory recess during both inhalation and exhalation, thus potentially increasing odorant absorption on olfactory epithelium [73]. In addition to differences in width and size, bats also display considerable variation in shape and orientation of external nares (Fig 1). While linear measurements such as those presented in this study can be reliable indicators of size variation, they are unlikely to completely reflect shape variation in shape [74]. Although geometric morphometric approaches are better at capturing shape data in morphological studies, the lack of consistent landmark features in soft tissues makes this technique difficult. Using advancements in three-dimensional imaging and reconstruction such as diceCT [75] or spiceCT [76], future work could investigate how this morphological variation might influence nasal airflow and thereby olfactory behavior.

Molossids (free-tailed bats) have the widest nostrils compared to other insectivores, even when accounting for differences in body size. Larger and wider nostrils may be advantageous for these high, fast flying species with high respiratory demand [77]. Molossids are also known for their strong odors; males of many free-tailed bats species (including all four molossid species used in this study) develop gular-thoracic glands that may be used to mark females or roosting sites [78,79]. It is possible that even if these species are unlikely to be using olfaction as a sensory cue while foraging, they may use olfactory cues to find potential mates, or as homing cues during migration, as observed in some species of seabirds [80–82].

Nose and nostril morphology are also influenced by respiratory and thermoregulatory demands [77], though how these demands interact with sniffing and olfaction is still unclear. In bats, the respiratory cycle is closely related to the wing-beat cycle, and echolocation pulses are generally emitted during expiration [83–85]. Sniffing, or bouts of increased air intake, is often associated with exposure to olfactory stimuli. Mammals, including rodents, moles, and bats will increase their sniff rates in response to olfactory stimuli [26,86]. Sniffing has only been rigorously testing in stationary bats, so it is still unknown how they balance olfactory inputs with respiratory demands, or how much they are able to sniff while flying.

Several experimental studies have demonstrated the importance of stereo-olfaction for scent-tracking in rodents [11,12,38], which are comparable in size to some bat species. Across all bat species in this study, the average inner nostril width ratio was 7.6, compared to 6.0 (from [31]) and 7.0 (this study, Table 3) for rodents. Among the bat species in this study, insectivorous molossid and vespertilionid bats had wider relative nostril widths even compared to rodents (Table 3). Studies on the computational fluid dynamics of airflow during sniffing in dogs and humans suggests that extreme wide nostrils are not necessary to take advantage of separate sampling areas [13,87,88]. During inspiration, air in the vicinity of the nostril is drawn towards the naris, creating a hemispherical region in front of the naris called the ‘reach’ of the nostril. In canines, the reach of a nostril is approximately 1 cm, which is smaller than the inter-nostril separation, indicating that each nostril is sampling air from spatial separate regions [87]. In humans, each nostril can sample information from areas that are separated by about 3.5 cm [13], which is wide enough to span the boundary of a scent plume (which can be within 10 mm [89]). Similar computational studies have not been done in rodents or bats, so it is unknown how these values might scale down to smaller animals. Narrow nostril widths in nectar feeding species does not preclude these species from using bilateral sampling mechanisms for olfactory localization, but wider widths in insectivorous bats suggest they may use olfactory tropotaxis more than expected.

**Table 3:** Average inner nostril width ratios (INWR) and standard error (SE) for a sample of mammalian taxa.

Taxa	INWR	± SE	Source
Insectivores			
Marsupials	5.1	0.69	Stoddart 1979
Tree shrews			
Rodents	6.0	0.41	Stoddart 1979
Rodents	7.0	0.40	this study
*Rattus rattus*	6.6	1.22	this study
*Mus musculus*	7.4	0.71	this study
Bats	7.6	0.45	this study
Molossidae	4.5	0.78	this study
Mormoopidae	7.0	0.44	this study
Phyllostomidae	9.5	0.48	this study
Vespertilionidae	4.9	0.23	this study

The relationship between morphology and olfactory ecology in bats is complicated by the tangential interactions between breathing, feeding, and echolocating, which can lead to compromises in the various physiological and mechanical parameters of the nose and rostrum. To our knowledge, this is the first study to evaluate the role of ecology in shaping the morphology of an external sensory character, using modern phylogenetic comparative methods. We found that nectar eating bat species have narrower nostrils than insect-eating species, and that nasal echolocation may impose constraints on tropotactic mechanisms for olfactory tracking in bats. Alternatively, the results also indicate that some insectivorous bats, like the Molossidae, may rely upon stereo-olfaction more than expected. Pairing morphology and physiological studies of olfaction with behavioral studies quantifying the patterns of olfactory tracking in bats will provide more insight into how bats integrate olfactory information while foraging.

## Supporting information

S1 Fig Boxplots representing variation in PC1 from the full dataset. a. Diet, b. foraging habitat, c. foraging mode, d. echolocation mode.
(TIF)

S1 Table Summary of species metadata. Variables used in this study, organized by species: sample size, body mass, diet category, foraging habitat, foraging mode, migration type, echolocation mode, flight speed, wing loading, and aspect ratio (where data was available).
(PDF)

S1 Appendix Museum specimen details and raw measurement data. (XLSX)

S1 File Comparisons of museum specimens and live animals. Figure A. Percent change between live and museum specimens for each morphological character. Figure B. Percent change between live and museum specimens for each morphological character. Figure C. Percent change between live and museum specimens for each species.
(PDF)

S2 File Phylogenetic signals for the morphological variables. Table A. Phylogenetic signal for each of the various morphometric measurements, using the full dataset consisting of all 40 species. Table B. Phylogenetic signal for each of the various morphometric measurements, from species within the family Phyllostomidae (n = 22).
(PDF)

S3 File Biplots and loadings from a phylogenetic principal component analysis. Figure A. Biplot of variable loadings from a phylogenetic principal component analysis on the full species dataset (n = 40). Table A. Loadings and percent variance explained by each PC axis, obtained using a phylogenetic principal component analysis on the full dataset (n = 40). Figure B. Biplot of variable loadings from a phylogenetic principal component analysis on species in the family Phyllostomidae (n = 22). Table B. Loadings and percent variance explained by each PC axis, obtained using a phylogenetic principal component analysis on species within Phyllostomidae (n = 22).
(PDF)

S4 File Summary of model outputs from phylogenetic generalized least squares regression analysis. Table A. Summary of outputs from phylogenetic generalized least squares regression analysis on principal component (PC) 1 and ecological variables, with body mass (BM) as a size covariate. Table B. Summary of outputs from phylogenetic generalized least squares regression analysis on principal component (PC) 1 and ecological variables, with forearm (FA) as a size covariate. Table C. Summary of outputs from phylogenetic generalized least squares regression analysis on principal component (PC) 2 and ecological variables, with body mass (BM) as a size covariate. Table D. Summary of outputs from phylogenetic generalized least squares regression analysis on principal component (PC) 2 and ecological variables, with forearm (FA) as a size covariate.
(PDF)

S5 File Boxplots and model outputs from a phylogenetic generalized least squares regression analysis for Phyllostomidae only. Figure A. Boxplots representing variation in phylogenetic Principal Component (PC) 1 (top) and PC 2 (bottom) for species within the species Phyllostomidae across diet categories. Figure B. Boxplots representing variation in phylogenetic Principal Component (PC) 1 (top) and PC 2 (bottom) for species within the species Phyllostomidae across foraging habitat categories. Table A. Summary of outputs from phylogenetic generalized least squares regression analysis on principal components and ecological variables for species within the family Phyllostomidae (n = 22 species), using body mass (BM) as a covariate. Table B. Summary of outputs from phylogenetic generalized least squares regression analysis on principal components and ecological variables for species within the family Phyllostomidae (n = 22 species), using forearm (FA) as a covariate.
(PDF)

S6 File Scatterplots and model outputs from phylogenetic generalized least squares regressions on quantitative flight traits. Figure A. Phylogenetic principal component (PC) 1 (top) and PC2 (bottom) plotted against log average speed for a subset of dataset (n = 21 species). Figure B. Phylogenetic principal component (PC) 1 (top) and PC2 (bottom) plotted against log wing loading for a subset of dataset (n = 25 species). Figure C. Phylogenetic principal component (PC) 1 (top) and PC2 (bottom) plotted against log aspect ratio for a subset of dataset (n = 35 species). Table A. Summary of outputs from phylogenetic generalized least squares regression analysis on principal components and log flight speed (n = 21 species). Table B. Summary of outputs from phylogenetic generalized least squares regression analysis on principal components and log wing loading (n = 25 species). Table C. Summary of outputs from phylogenetic generalized least squares regression analysis on principal components and log aspect ratio (n = 35 species).
(PDF)
