# Supplementary figures and images for: Role of ecology in shaping external nasal morphology in bats and implications for olfactory tracking
Source: PLoS One. 2020 Jan 8;15(1):e0226689. doi: 10.1371/journal.pone.0226689 (PMC6948747; doi:10.1371/journal.pone.0226689)

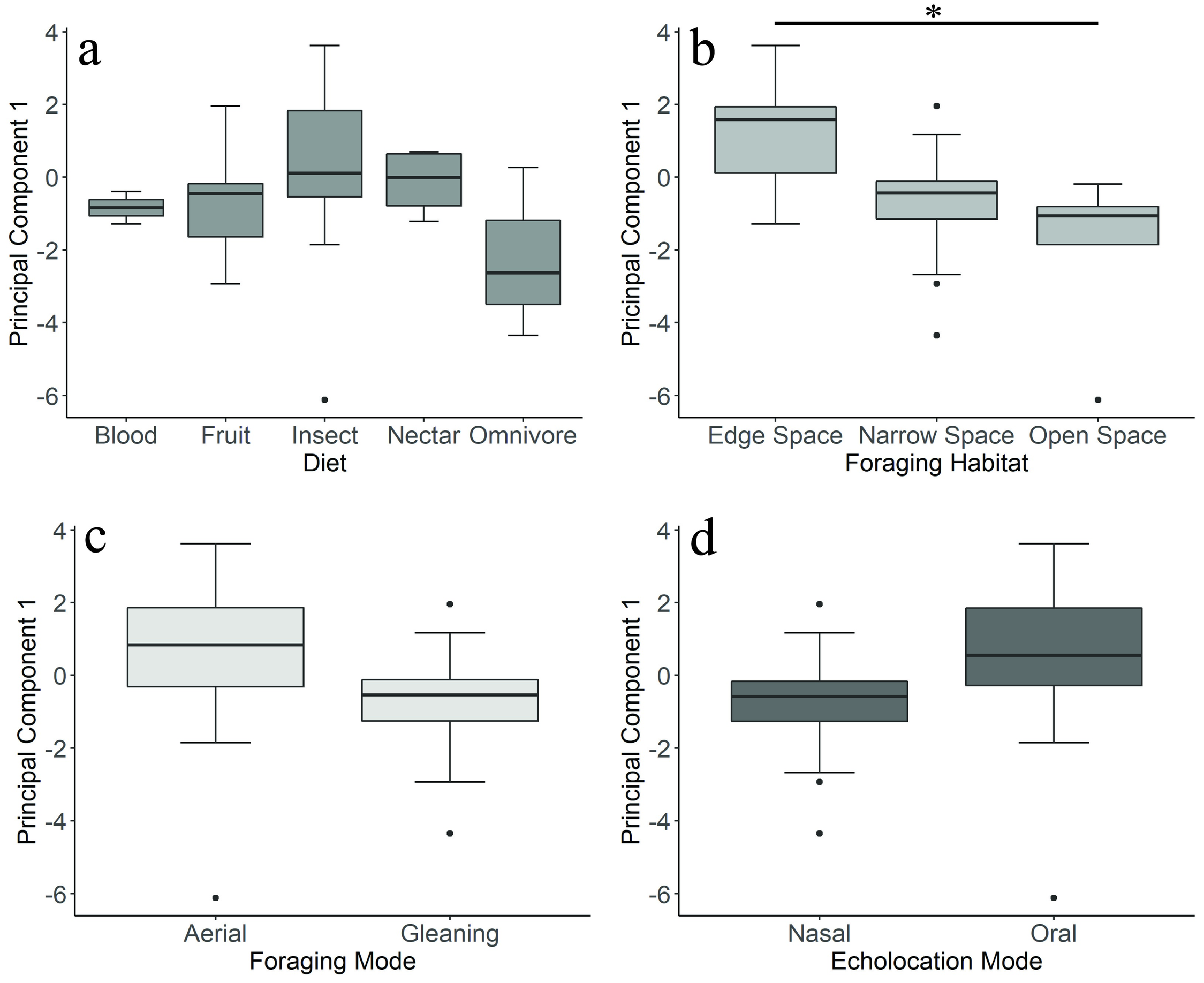

Supplement: S1 Fig — a. Diet, b. foraging habitat, c. foraging mode, d. echolocation mode. (TIF) [file pone.0226689.s001.tif]
