# Supplementary material for: Role of ecology in shaping external nasal morphology in bats and implications for olfactory tracking
Source: PLoS One. 2020 Jan 8;15(1):e0226689. doi: 10.1371/journal.pone.0226689 (PMC6948747; doi:10.1371/journal.pone.0226689)
Supplement: S1 Table — Variables used in this study, organized by species: sample size, body mass, diet category, foraging habitat, foraging mode, migration type, echolocation mode, flight speed, wing loading, and aspect ratio (where data was available). (PDF) [file pone.0226689.s002.pdf]

Taxon	Sample Size	Body Mass (g)[1]	Diet Category	Forage Habitat	Forage Mode	Migrate?	Echo Mode	Flight Speed (m/s)	Wing Load (g/cm^2)	Aspect Ratio
Eumops perotis	6	50.9	Insect[2]	Open	Aerial	No	Oral	6.57[3]	0.27[3]	9.98[3]
Molossus rufus	10	32[4]	Insect	Open	Aerial	No	Oral			
Nyctinomops macrotis	4	20.8	Insect[5]	Open	Aerial	No	Oral	8.94[5]		9.71[5]
Tadarida brasiliensis	10	12.2	Insect	Open	Aerial	Yes	Oral	5.19[3]	0.116[6]	8.6[6]
Mormoops megalophylla	10	16	Insect	Edge	Aerial	No	Oral	5.26[7]		7.5[8]
Pteronotus gymnonotus	10	13.6	Insect	Edge	Aerial	No	Oral			8.7[8]
Pteronotus parnellii	10	19.5	Insect[9]	Narrow	Aerial	No	Oral	4.87[7]		6.48[10]
Pteronotus personatus	10	5.6	Insect[11]	Edge	Aerial	No	Oral	4.31[7]		
Anoura geoffroyi	9	15	Nectar[12]	Narrow	Gleaning	No	Nasal			6.5[12]
Artibeus jamaicensis	10	41.6	Fruit[13]	Narrow	Gleaning	No	Nasal	3.75[14]	0.37[15]	6.4[8]
Artibeus lituratus	10	59.3	Fruit	Narrow	Gleaning	No	Nasal		0.39[15]	6.25[10]
Artibeus phaeotis	10	11.7	Fruit[16]	Narrow	Gleaning	No	Nasal		0.10[17]	6.33[17]
Carollia perspicillata	10	19.1	Fruit[18]	Narrow	Gleaning	No	Nasal	4.00[19]	0.25[15]	6.22[10]
Chiroderma trinitatum	5	23.6	Fruit	Narrow	Gleaning	No	Nasal			6.24[10]
Chiroderma villosum	3	25	Fruit[20]	Narrow	Gleaning	No	Nasal		0.14[17]	6.37[10]
Desmodus rotundus	10	33	Blood[21]	Edge	Gleaning	No	Nasal	3.75[22]	0.16[17]	6.73[23]
Diphylla ecaudata	9	28.11	Blood[24]	Edge	Gleaning	No	Nasal			
Glossophaga soricina	10	9.9	Nectar[25,26]	Narrow	Gleaning	No	Nasal	4.8[27]	0.09[17]	6.47[10]
Leptonycteris yerbabuenae	10	22.24	Nectar[28]	Narrow	Gleaning	Yes	Nasal	7.6[29]	0.16[30]	7.10[30]
Lonchophylla handleyi	9	17[31]	Nectar[32]	Narrow	Gleaning	No	Nasal			
Lonchorhina aurita	2	15.3	Insect[33]	Narrow	Gleaning	No	Nasal			
Macrotus waterhousii	10	16.1	Insect[34]	Narrow	Gleaning	No	Nasal			9.00[8]
Micronycteris megalotis	10	13.8	Insect[35]	Narrow	Gleaning	No	Nasal			5.74[10]
Mimon crenulatum	2	6.4	Insect	Narrow	Gleaning	No	Nasal			6.45[10]
Phyllostomus discolor	11	41.4	Omnivore[36]	Narrow	Gleaning	No	Nasal		0.15[36]	6.93[10]
Phyllostomus hastatus	10	91.1	Omnivore[37]	Narrow	Gleaning	No	Nasal	8.0[14]	0.20[38]	6.80[38]
Sturnira lilium	10	20.2	Fruit[39]	Narrow	Gleaning	No	Nasal	4.17[40]	0.13[17]	6.27[10]
Uroderma bilobatum	10	16.2	Fruit[41]	Narrow	Gleaning	No	Nasal		0.10[17]	6.3[10]
Vampyressa bidens	10	11.8	Fruit[42]	Narrow	Gleaning	No	Nasal		0.11[42]	6.38[10]
Vampyressa pusilla	2	8.6	Fruit[43]	Narrow	Gleaning	No	Nasal		0.11[43]	5.88[43]
Antrozous pallidus	10	22.2	Omnivore[44]	Narrow	Gleaning	No	Oral	4.00[45]	0.12[6]	6.55[6]
Eptescicus fuscus	9	17.3	Insect[46]	Edge	Aerial	No	Oral	5.15[45]	0.09[6]	7.06[6]
Lasiurus borealis	10	12.3	Insect	Edge	Aerial	Yes	Oral	3.53[47]	0.08[6]	7.55[6]
Lasiurus cinereus	6	26.8	Insect[48]	Open	Aerial	Yes	Oral	5.05[45]	0.13[6]	8.25[6]

Lasiurus seminolus	7	9.87	Insect	Edge	Aerial	No	Oral			6.7[8]
Myotis nigricans	10	4.25[49]	Insect	Edge	Aerial	No	Oral	2.41[50]	0.07[8]	6.5[8]
Myotis velifer	11	9.78	Insect[51]	Edge	Aerial	No	Oral	4.51[45]	0.06[6]	6.73[6]
Myotis yumanensis	8	5.15	Insect	Edge	Aerial	No	Oral	3.88[45]	0.05[51]	6.45[45]
Pipstrellus subflavus	6	5.7	Insect	Edge	Aerial	No	Oral	4.35[47]	0.09[6]	6.92[6]
Rhogeessa tumida	8	4.58	Insect	Edge	Aerial	No	Oral		0.08[17]	6.2[17]

S1 Table. Variables used in this study, organized by species: sample size, body mass, diet category, foraging habitat, foraging mode, migration type, echolocation mode, flight speed, wing loading, and aspect ratio (where data was available).

Literature Cited:

1. Jones KE, Bielby J, Cardillo M, Fritz SA, O'Dell J, Orme CDL, et al. PanTHERIA: a species-level database of life history, ecology, and geography of extant and recently extinct mammals. Michener WK, editor. Ecology. 2009;90: 2648–2648. doi:10.1890/08-1494.1
2. Best TL, Kiser WM, Freeman PW, Kiser WM, Freeman PW. Eumops perotis. Mamm Species. 1996;534: 1–8.
3. Vaughan TA. Morphology and Flight Characteristics of Molossid Bats. J Mammal. 1966;47: 249–260. doi:10.2307/1378121
4. Reid F. A Field Guide to the Mammals of Central America and Southeast Mexico. New York, NY: Oxford University Press; 2009.
5. Milner J, Jones C, Jones JK. *Nyctinomops macrotis*. Mamm Species. 1990;351: 1--. Available: http://www.jstor.org/stable/3504187
6. Farney J, Fleharty ED. Aspect Ratio, Loading, Wing Span, and Membrane Areas of Bats. J Mammal. 1969;50: 362–367. doi:10.2307/1378361
7. Kennedy ML, Price PK, Fuller OS. Flight Speeds of Five Species of Neotropical Bats. Southwest Nat. 1977;22: 401–404.
8. Norberg UM, Rayner JMV. Ecological morphology and flight in bats (Mammalia; Chiroptera): wing adaptations, flight performance, foraging strategy and echolocation. Philos Trans R Soc B, Biol Sci. 1987;316: 335–427.
9. Herd RM. *Pteronotus parnellii*. Mamm Species. 1983;209: 1–5. doi:10.1644/826.1.Key
10. Marinello MM, Bernard E. Wing morphology of Neotropical bats: a quantitative and qualitative analysis with implications for habitat use. Can J Zool. 2014;92: 141–147. doi:10.1139/cjz-2013-0127
11. Antonio de la Torre J, Medellín RA. *Pteronotus personatus* (Chiroptera : Mormoopidae). Mamm Species. 2010;42: 244–250.
12. Ortega J, Alarcón-D I. *Anoura geoffroyi*. Mamm Species. 2008;818: 1–7. doi:10.1644/818.1.Key
13. Ortega J, Castro-Arellano I. *Artibeus jamaicensis*. Mamm Species. 2001;6662: 1–9.

14. Morrison DW. Flight speeds of some tropical forest bats. Am Midl Nat. 1980;104: 189–192. doi:10.2307/2424971
15. Stockwell EF. Morphology and flight manoeuvrability in New World leaf-nosed bats (Chiroptera : Phyllostomidae). J Zool. 2001;254: 505–514.
16. Timm RM. *Artibeus phaeotis*. Mamm Species. 1985;235: 1–6. doi:10.1126/science.95.2469.427-b
17. Lawlor TE. Aerodynamic Characteristics of Some Neotropical Bats. J Mammal. 1973;54: 71–78. Available: http://www.jstor.org/stable/1378873
18. Cloutier D, Thomas DW. *Carollia perspicillata*. Mamm Species. 1992;417: 1–9. doi:10.1644/826.1.Key
19. von Busse R, Swartz SM, Voigt CC. Flight metabolism in relation to speed in Chiroptera: testing the U-shape paradigm in the short-tailed fruit bat *Carollia perspicillata*. J Exp Biol. 2013;216: 2073–2080. doi:10.1242/jeb.081760
20. Nogueira MR, Peracchi AL. Fig-seed predation by 2 species of *Chiroderma*: discovery of a new feeding strategy in bats. J Mammal. 2005;84: 225–233. doi:10.1644/1545-1542(2003)084<0225:fspbso>2.0.co;2
21. Greenhall AM, Joermann G, Schmidt U, Seidel MR. *Desmodus rotundus*. Mamm Species. 1983;202: 1–6.
22. Sánchez-Hernández C, Romero-Almaraz M de L, Wooten MC, Schnell GD, Kennedy ML. Speed in flight of Common Vampire Bats (*Desmodus rotundus*). Southwest Nat. 2006;51: 422–425. doi:10.1894/0038-4909(2006)51[422:sifocv]2.0.co;2
23. Struhsaker TT. Morphological Factors Regulating Flight in Bats. J Mammal. 1961;42: 152. doi:10.2307/1376823
24. Elizalde-Arellano C, López-Vidal JC, Arroyo-Cabrales J, Medellin RA, Laundré JW. Food sharing behavior in the hairy-legged vampire bat. Acta Chiropterologica. 2000;9: 314–319. doi:10.3161/1733-5329(2007)9[314:fsbith]2.0.co;2
25. Alvarez J, Willig MR, Jones JKJ, Webster WD. *Glossophaga soricina*. Source Mamm Species. 1991;379: 1–7. doi:10.1644/826.1.Key
26. Clare EL, Goerlitz HR, Drapeau VA, Holderied MW, Adams AM, Nagel J, et al. Trophic niche flexibility in *Glossophaga soricina*: How a nectar seeker sneaks an insect snack. Funct Ecol. 2014;28: 632–641. doi:10.1111/1365-2435.12192
27. Akins JB, Kennedy ML, Schnell GD, Sánchez-Hernández C, de Lourdes Romero-Almaraz M, Wooten MC, et al. Flight speeds of three species of Neotropical bats: *Glossophaga soricina*, *Natalus stramineus*, and *Carollia subrufa*. Acta Chiropterologica. 2007;9: 477–482. doi:10.3161/1733-5329(2007)9[477:FSOTSO]2.0.CO;2
28. Ayala-Berdon J, Rodríguez-Peña N, García Leal C, Stoner KE, Schondube JE. Sugar gustatory thresholds and sugar selection in two species of Neotropical nectar-eating bats. Comp Biochem Physiol - A Mol Integr Physiol. 2013;164: 307–313. doi:10.1016/j.cbpa.2012.10.019
29. Sahley CT, Horner MA, Fleming TH. Flight Speeds and Mechanical Power Outputs of the Nectar-Feedint Bat, *Leptonycteris curasoae (*Phyllostomidae: Glossophaginae). J Mammal. 2006;74: 594–600. doi:10.2307/1382278
30. Håkansson J, Hedenström A, Winter Y, Johansson LC. The wake of hovering flight in bats. J R Soc Interface. 2015;12: 20150357. doi:10.1098/rsif.2015.0357
31. Solari S, Pacheco V, Vivar E. New distribution records of Peruvian bats. Rev Peru Biol. 1999;6: 152–159.
32. Datzmann T, Von Helversen O, Mayer F. Evolution of nectarivory in phyllostomid bats (Phyllostomidae, Gray, 1825,

Chiroptera: Mammalia). BMC Evol Biol. 2010;10: 165. doi:10.1186/1471-2148-10-165
33. Lassieur S, Wilson DE. *Lonchorhina aurita*. Mamm Species. 1989;347: 1–4. Available: https://repository.si.edu/bitstream/handle/10088/4718/VZ_dew3.pdf
34. Anderson S. Macrotus waterhousii. Mamm Species. 1969;1: 1–4.
35. Alonso-Mejía A, Medellín RA. *Micronycteris megalotis*. Mamm Species. 1991;376: 1–6. doi:10.1126/science.95.2469.427-b
36. Kwiecinski GG. *Phyllostomus discolor*. Mamm Species. 2006;801: 1–11. doi:10.1644/826.1.Key
37. Santos M, Aguirre LF, Vazquez LB, Ortega J, Lacepede P. *Phyllostomus hastatus*. Mamm Species. 2003;722: 1–6. doi:10.1644/0.722.1/2600811
38. Stern AA, Kunz TH, Bhatt SS. Seasonal Wing Loading and the Ontogeny of Flight in *Phyllostomus hastatus* (Chiroptera: Phyllostomidae). J Mammal. 1997;78: 1199–1209.
39. Mello MAR, Kalko EK V., Silva WR. Diet and Abundance of the Bat *Sturnira lilium* (Chiroptera) in a Brazilian Montane Atlantic Forest. J Mammal. 2008;89: 485–492. doi:10.1644/06-MAMM-A-411R.1
40. Loayza AP, Loiselle BA. Preliminary information on the home range and movement patterns of *Sturnira lilium* (Phyllostomidae) in a naturally fragmented landscape in Bolivia. Biotropica. 2008;40: 630–635. doi:10.1111/j.1744-7429.2008.00422.x
41. Baker RJ, Clark CL. *Uroderma bilobatum*. Mamm Species. 1987;279: 1–4. doi:10.1007/sl0869-007-9037-x
42. Lee BTE, Scott JB, Marcum MM, Dobson V, Bat BY. *Vampyressa bidens*. Mamm Species. 2001;28: 1–3. doi:10.2307/0.684.1/2600180
43. Lewis SE, Wilson DE. *Vampyressa pusilla*. Mamm Species. 1987;292: 1–5. Available: http://www.jstor.org/stable/3503881
44. Aliperti JR, Kelt DA, Heady III PA, Frick WF. Using behavioral and stable isotope data to quantify rare dietary plasticity in a temperate bat. J Mammal. 2017;XX: 1–10. doi:10.1093/jmammal/gyw196
45. Hayward B, Davis R. Flight speeds in western bats. Am Soc Mammal. 1964;45: 7. doi:10.2307/1376986
46. Agosta SJ. Habitat use, diet and roost selection by the big brown bat (*Eptesicus fuscus*) in North America: A case for conserving an abundant species. Mamm Rev. 2002;32: 179–198. doi:10.1046/j.1365-2907.2002.00103.x
47. Patterson AP, Hardin JW. Flight speeds of five species of Vespertilionid bats. J Mammal. 1969;50: 152–153. doi:10.2307/30054812
48. Reimer JP, Baerwald EF, Barclay RMR. Diet of Hoary (*Lasiurus cinereus*) and Silver-haired (*Lasionycteris noctivagans*) Bats While Migrating Through Southwestern Alberta in Late Summer and Autumn. Am Midl Nat. 2010;164: 230–237. doi:10.1674/0003-0031-164.2.230
49. Wilson DE, LaVal RK. *Myotis nigricans*. Mamm Species. 1974; 1. doi:10.2307/3503847
50. Voigt CC. Bat flight with bad wings: is flight metabolism affected by damaged wings? J Exp Biol. 2013;216: 1516–1521. doi:10.1242/jeb.079509
51. Braun JK, Yang B, Gonzalez-Perez SB, Mares MA. *Myotis yumanensis* (Chiroptera: Vespertilionidae). Mamm Species.

2015;47: 1–14. doi:10.1093/mspecies/sev001
