# Supplementary material for: Role of ecology in shaping external nasal morphology in bats and implications for olfactory tracking
Source: PLoS One. 2020 Jan 8;15(1):e0226689. doi: 10.1371/journal.pone.0226689 (PMC6948747; doi:10.1371/journal.pone.0226689)
Supplement: S1 File — Figure A. Percent change between live and museum specimens for each morphological character. Figure B. Percent change between live and museum specimens for each morphological character. Figure C. Percent change between live and museum specimens for each species. (PDF) [file pone.0226689.s004.pdf]

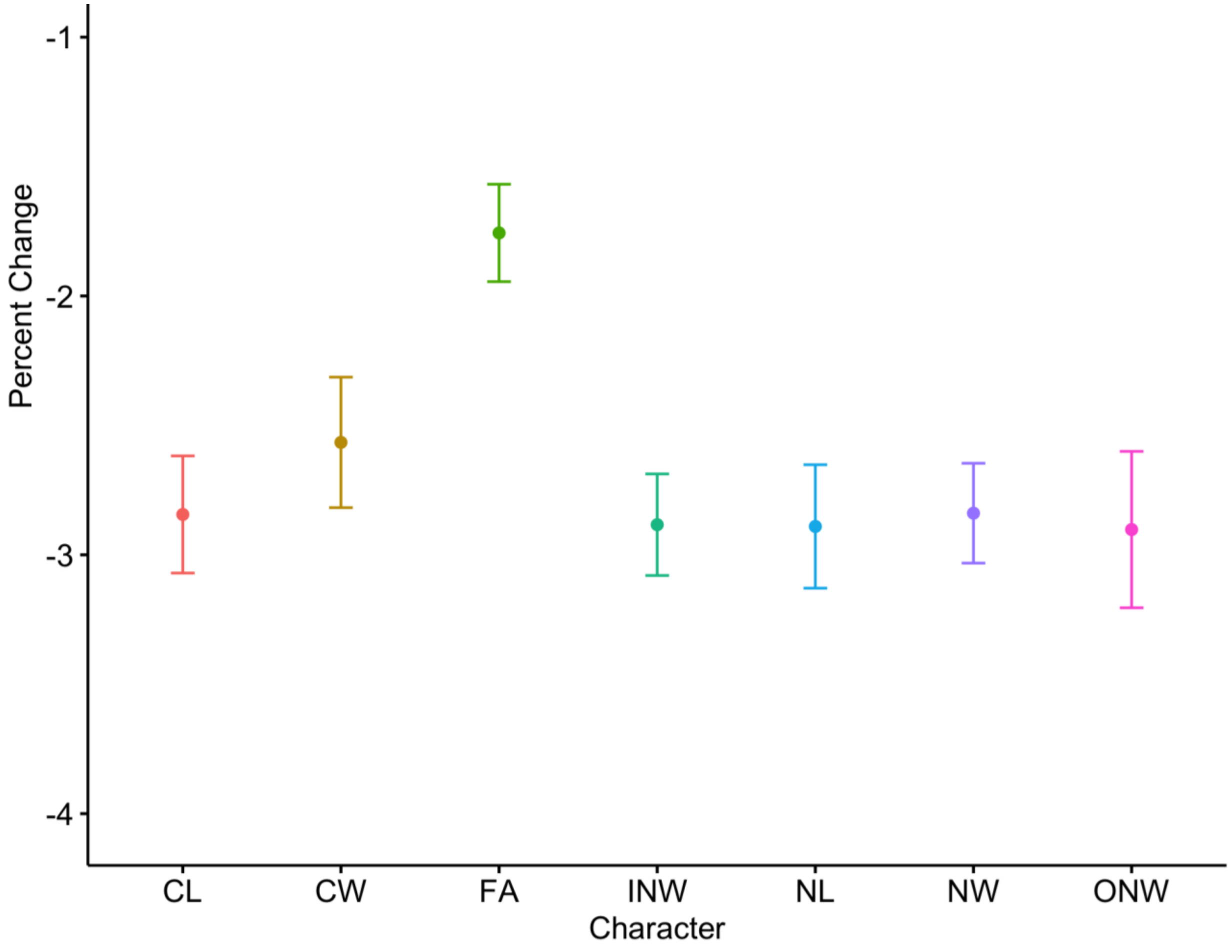

Figure A. Percent change between live and museum specimens for each morphological character. Percent change in forearm was significantly difference compared to all other measurements except CW (post-hoc pairwise comparisons, α = 0.05). CL, cranial length, CW: cranial width, FA: forearm, INW: inner nostril width, NL: nose length, NW: nose width, ONW: outer nostril width.

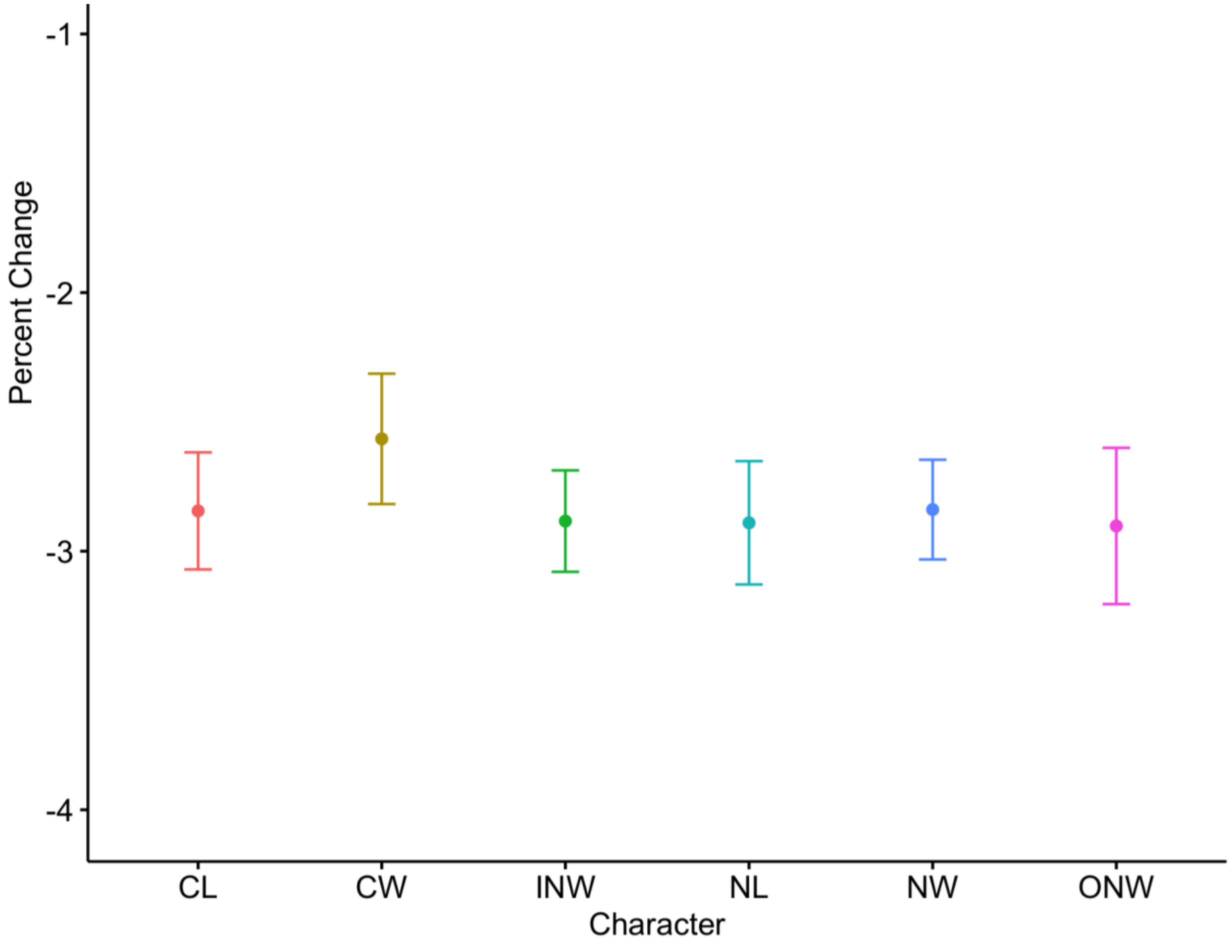

Figure B. Percent change between live and museum specimens for each morphological character. When forearm was excluded from the analysis, there was no significant difference in percent change across morphological measurements (one-way ANOVA, F = 0.289, P = 0.917). Abbreviations: CL, cranial length, CW: cranial width, INW: inner nostril width, NL: nose length, NW: nose width, ONW: outer nostril width.

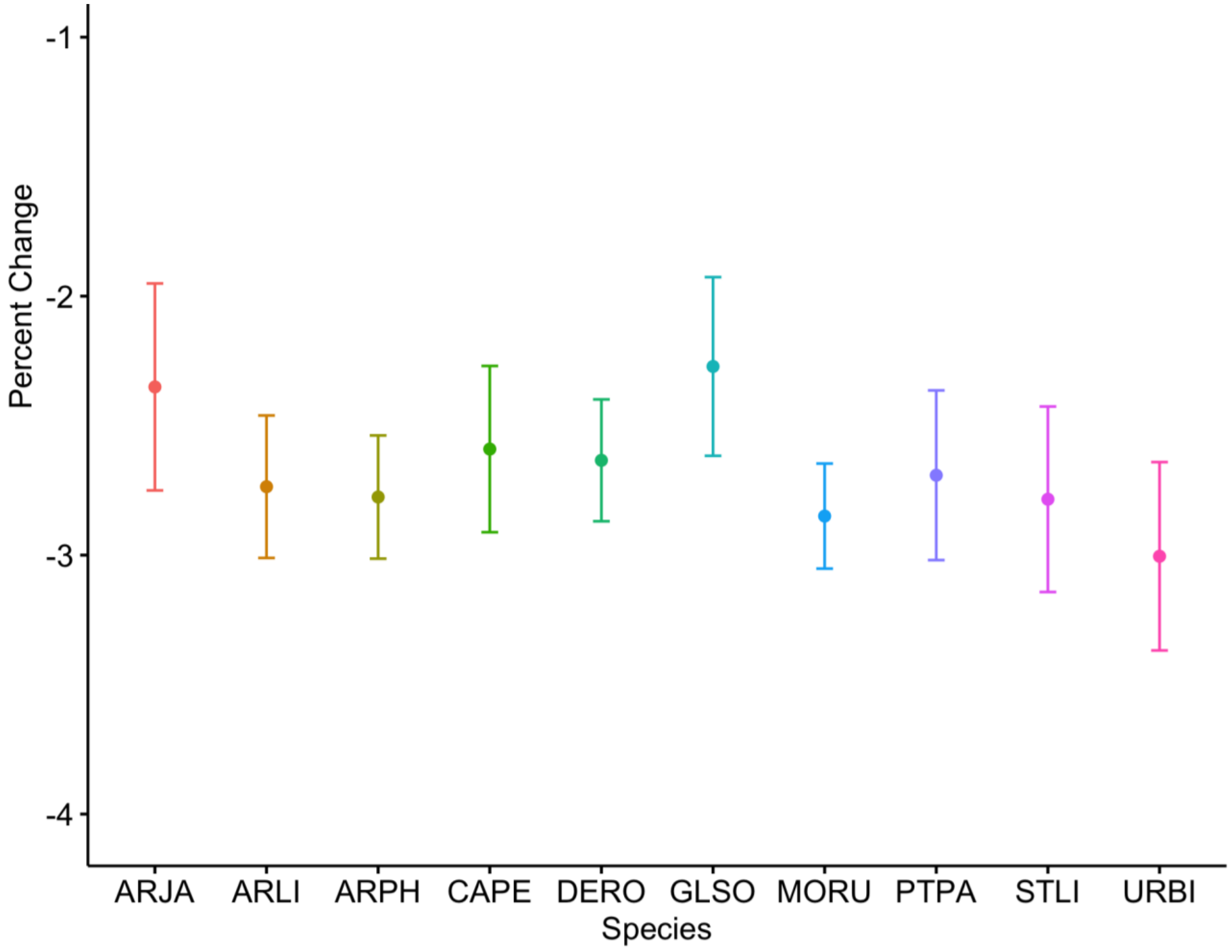

Figure C. Percent change between live and museum specimens for each species. There was no significant difference in percent change across different species (one-way ANOVA, $F = 0.501$, $P = 0.868$). Abbreviations: ARJA: *Artibeus jamaicensis*, ARLI: *A. lituratus*, ARPH: *A. phaeotis*, CAPE: *Carollia perspicillata*, DERO: *Desmodus rotundus*, GLSO: *Glossophaga soricina*, MORU: *Molossus rufus*, PTPA: *Pteronotus parnellii*, STLI: *Sturnira lilium*, URBI: *Uroderma bilobatum*.
