# Supplementary material for: Role of ecology in shaping external nasal morphology in bats and implications for olfactory tracking
Source: PLoS One. 2020 Jan 8;15(1):e0226689. doi: 10.1371/journal.pone.0226689 (PMC6948747; doi:10.1371/journal.pone.0226689)
Supplement: S2 File — Table A. Phylogenetic signal for each of the various morphometric measurements, using the full dataset consisting of all 40 species. Table B. Phylogenetic signal for each of the various morphometric measurements, from species within the family Phyllostomidae (n = 22). (PDF) [file pone.0226689.s005.pdf]

Table A. Phylogenetic signal for each of the various morphometric measurements, using the full dataset consisting of all 40 species.

Character	Estimated Pagel's λ	*P*-value	Estimated Blomberg's K	*P*-value
**Average Mass**	0.045	0.813	0.509	0.313
**INW**	0.999	*<0.001*	1.594	*<0.001*
**ONW**	0.999	*0.002*	0.825	*<0.001*
**NL**	0.999	*0.018*	0.725	*0.016*
**NW**	0.872	*0.005*	0.769	*0.002*
**CL**	0.762	*<0.001*	0.845	*0.003*
**CW**	0.685	0.056	0.643	*0.046*
**FA**	0.384	0.356	0.567	0.073
**INWR**	0.999	*<0.001*	1.497	*<0.001*
**NareW**	0.399	*0.031*	0.666	*0.018*

Table B. Phylogenetic signal for each of the various morphometric measurements, from species within the family Phyllostomidae (n = 22).

Character	Estimated Pagel's λ	*P*-value	Estimated Blomberg's K	*P*-value
**Average Mass**	~ 0	1	0.669	0.574
**INW**	0.999	0.129	0.950	0.061
**ONW**	0.481	1	0.798	0.238
**NL**	0.752	0.329	0.863	0.112
**NW**	0.516	0.820	0.799	0.204
**CL**	~ 0	1	0.621	0.629
**CW**	~ 0	1	0.573	0.735
**FA**	0.168	0.860	0.709	0.397
**INWR**	0.991	0.087	0.975	*0.039*
**NareW**	~ 0	1	0.664	0.559
