# Supplementary material for: Role of ecology in shaping external nasal morphology in bats and implications for olfactory tracking
Source: PLoS One. 2020 Jan 8;15(1):e0226689. doi: 10.1371/journal.pone.0226689 (PMC6948747; doi:10.1371/journal.pone.0226689)
Supplement: S3 File — Figure A. Biplot of variable loadings from a phylogenetic principal component analysis on the full species dataset (n = 40). Table A. Loadings and percent variance explained by each PC axis, obtained using a phylogenetic principal component analysis on the full dataset (n = 40). Figure B. Biplot of variable loadings from a phylogenetic principal component analysis on species in the family Phyllostomidae (n = 22). Table B. Loadings and percent variance explained by each PC axis, obtained using a phylogenetic principal component analysis on species within Phyllostomidae (n = 22). (PDF) [file pone.0226689.s006.pdf]

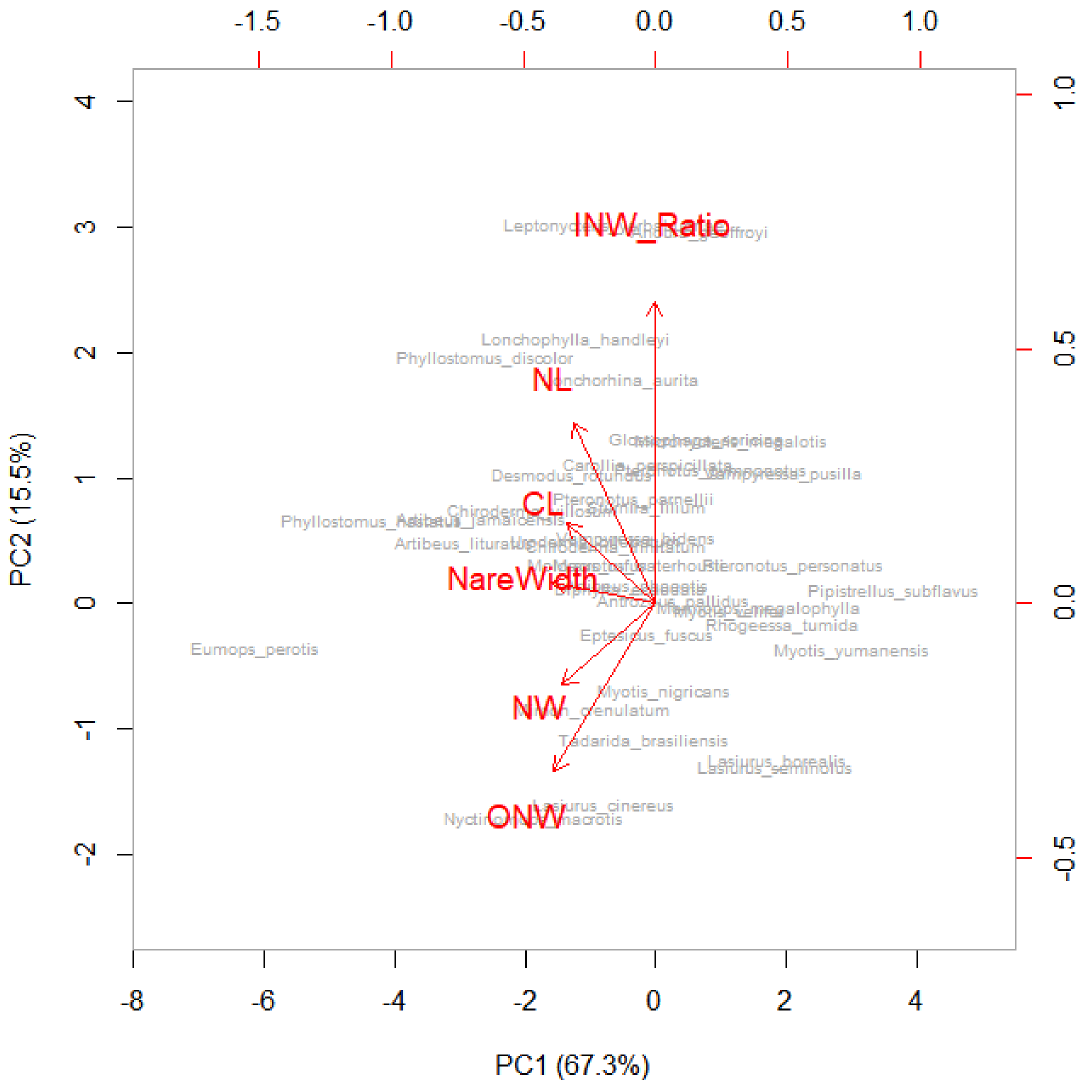

Figure A. Biplot of variable loadings from a phylogenetic principal component analysis on the full species dataset (n = 40).

Table A. Loadings and percent variance explained by each PC axis, obtained using a phylogenetic principal component analysis on the full dataset (n=40).

Character	PC1	PC2	PC3
**ONW**	-0.911	-0.375	0.050
**NL**	-0.708	0.390	0.587
**NW**	-0.897	-0.195	-0.123
**CL**	-0.906	0.205	-0.171
**INWR**	-0.018	0.906	0.412
**NareW**	-0.915	0.048	-0.282
**Percent variation**	67.3	15.5	10.9

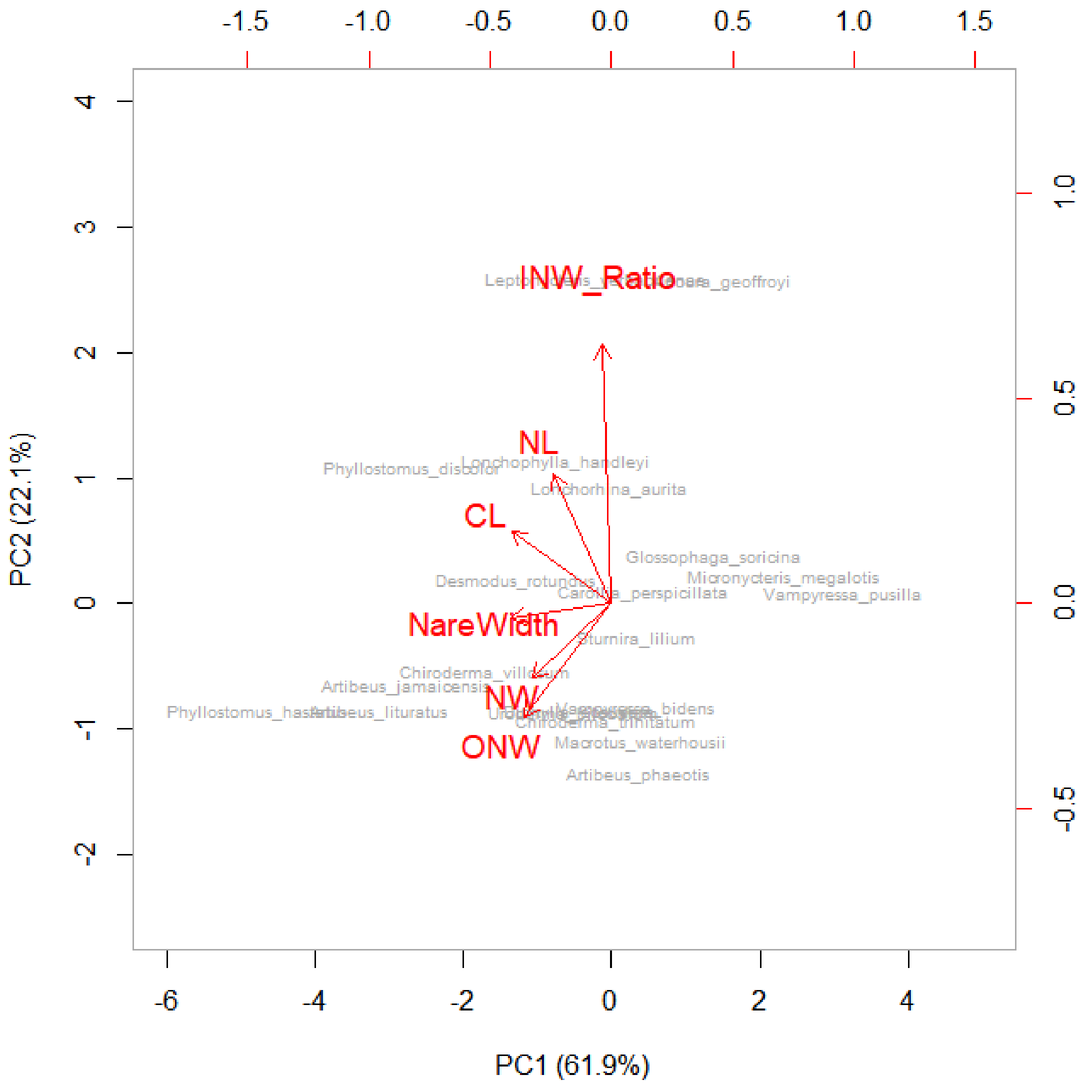

Figure B. Biplot of variable loadings from a phylogenetic principal component analysis on species in the family Phyllostomidae (n = 22).

Table B. Loadings and percent variance explained by each PC axis, obtained using a phylogenetic principal component analysis on species within Phyllostomidae (n=23).

Character	PC1	PC2	PC3
**ONW**	-0.895	-0.409	-0.020
**NL**	-0.602	0.474	0.637
**NW**	-0.828	-0.271	0.074
**CL**	-0.922	0.236	-0.156
**INWR**	-0.099	0.947	-0.272
**NareW**	-0.939	-0.050	-0.187
**Percent variation**	61.9	22.1	8.6
