# Supplementary material for: Role of ecology in shaping external nasal morphology in bats and implications for olfactory tracking
Source: PLoS One. 2020 Jan 8;15(1):e0226689. doi: 10.1371/journal.pone.0226689 (PMC6948747; doi:10.1371/journal.pone.0226689)
Supplement: S4 File — Table A. Summary of outputs from phylogenetic generalized least squares regression analysis on principal component (PC) 1 and ecological variables, with body mass (BM) as a size covariate. Table B. Summary of outputs from phylogenetic generalized least squares regression analysis on principal component (PC) 1 and ecological variables, with forearm (FA) as a size covariate. Table C. Summary of outputs from phylogenetic generalized least squares regression analysis on principal component (PC) 2 and ecological variables, with body mass (BM) as a size covariate. Table D. Summary of outputs from phylogenetic generalized least squares regression analysis on principal component (PC) 2 and ecological variables, with forearm (FA) as a size covariate. (PDF) [file pone.0226689.s007.pdf]

Model: PC1	AICc	ΔAICc	λ Est	F-stat	Model P-value	Adjusted R^2	P-values					
							Body Mass	Diet	Forage Habitat	Forage Mode	Echo Mode	Migrate Type
BM + FH + M	122.19	0.00	0.00	24.28	$1.11e^{-9}$	0.705	$5.18e^{-11}$	-	*0.034*	-	-	0.101
BM + FH	122.68	0.49	0.00	29.91	$6.98e^{-10}$	0.690	$7.43e^{-11}$	-	*0.039*	-	-	-
BM + FH + EM + M	123.86	1.68	0.00	19.59	$3.75e^{-6}$	0.705	$6.97e^{-11}$	-	*0.035*	-	0.333	0.102
BM + FH + EM	124.27	2.08	0.00	22.61	$2.71e^{-9}$	0.689	$9.93e^{-11}$	-	*0.040*	-	0.345	-
BM	124.38	2.19	0.55	57.64	$4.02e^{-9}$	0.592	$4.02e^{-9}$	-	-	-	-	-
BM + FH + FM + M	124.84	2.66	0.00	18.95	$5.62e^{-9}$	0.697	$9.43e^{-11}$	-	*0.037*	0.722	-	0.107
BM + FH + FM	125.16	2.97	0.00	21.92	$3.97e^{-9}$	0.682	$1.31e^{-10}$	-	*0.043*	0.728	-	-
BM + FH + FM + EM + M	125.85	3.66	0.00	16.37	$1.21e^{-8}$	0.728	$9.97e^{-11}$	-	*0.036*	0.720	0.223	0.098
BM + FH + FM + EM	126.26	4.07	0.00	18.06	$1.01e^{-8}$	0.686	$1.45e^{-10}$	-	*0.041*	0.727	0.235	-
BM + M	126.33	4.14	0.52	28.65	$3.04e^{-8}$	0.587	$5.38e^{-9}$	-	-	-	-	0.546
BM + EM	126.41	4.22	0.52	28.58	$3.13e^{-8}$	0.586	$5.47e^{-9}$	-	-	-	0.589	-
BM + FM	126.72	4.53	0.55	28.04	$3.87e^{-8}$	0.581	$6.39e^{-9}$	-	-	0.956	-	-
BM + FM + EM	128.24	6.05	0.45	19.32	$1.23e^{-8}$	0.585	$6.24e^{-9}$	-	-	0.930	0.349	-
BM + EM + M	128.52	6.33	0.49	18.90	$1.57e^{-8}$	0.579	$7.59e^{-9}$	-	-	-	0.589	0.571
BM + FM + M	128.80	6.62	0.52	18.58	$1.88e^{-7}$	0.575	$8.70e^{-9}$	-	-	0.950	-	0.554
BM + FM + EM + M	130.36	8.18	0.39	14.60	$4.15e^{-7}$	0.582	$7.55e^{-9}$	-	-	0.915	0.344	0.507
BM + D	133.34	11.15	0.55	10.84	$2.74e^{-6}$	0.558	$1.94e^{-8}$	0.894	-	-	-	-
BM + D + FH	133.96	11.77	0.00	11.48	$3.42e^{-7}$	0.653	$8.02e^{-10}$	0.880	0.085	-	-	-
BM + D + FH + M	134.31	12.12	0.00	10.80	$4.13e^{-7}$	0.668	$6.44e^{-10}$	0.871	0.077	-	-	0.130
BM + D + EM	134.88	12.69	0.37	9.68	$3.72e^{-6}$	0.572	$1.40e^{-8}$	0.894	-	-	0.264	-
BM + D + FH + EM	135.19	13.00	0.00	10.48	$5.69e^{-7}$	0.660	$8.25e^{-10}$	0.876	0.081	-	0.203	-
BM + D + M	135.77	13.58	0.50	9.05	$7.18e^{-6}$	0.553	$2.47e^{-8}$	0.897	-	-	-	0.508
BM + D + FM	135.80	13.61	0.45	9.15	$6.85e^{-6}$	0.556	$2.21e^{-8}$	0.897	-	0.507	-	-
BM + D + FH + FM	136.91	14.72	0.00	9.87	$1.06e^{-6}$	0.645	$1.33e^{-9}$	0.884	0.090	0.578	-	-
BM + D + EM + M	137.40	15.21	0.00	10.16	$1.24e^{-6}$	0.622	$2.10e^{-9}$	0.896	-	-	0.196	0.479
BM + D + FH + FM + M	137.46	15.27	0.00	9.43	$1.23e^{-6}$	0.660	$1.09e^{-9}$	0.876	0.082	0.570	-	0.135

BM + D + FM + EM	137.63	15.44	0.00	10.08	$1.35e^{-6}$	0.620	$2.23e^{-9}$	0.897	-	0.354	0.285	-
BM + D + FH + FM + EM	138.42	16.23	0.00	9.12	$1.72e^{-6}$	0.652	$1.41e^{-9}$	0.880	0.086	0.574	0.216	-
BM + D + FM + M	138.46	16.27	0.39	7.87	$1.52e^{-5}$	0.552	$2.74e^{-8}$	0.900	-	0.493	-	0.534
BM + D + FH + FM + EM + M	139.05	16.86	0.00	8.84	$1.88e^{-6}$	0.668	$1.14e^{-9}$	0.871	0.078	0.566	0.206	0.130
BM + D + FM + EM + M	140.27	18.08	0.00	8.77	$3.54e^{-6}$	0.614	$3.30e^{-9}$	0.899	-	0.358	0.289	0.460
~ 1	158.19	36.01	0.90	-	-	0.000	-	-	-	-	-	-

Table A. Summary of outputs from phylogenetic generalized least squares regression analysis on principal component (PC) 1 and ecological variables, with body mass (BM) as a size covariate. D: diet, FH: foraging habitat, FM: foraging mode, EM: echolocation mode, M: migratory type.

**Model: PC1**	**AICc**	**ΔAICc**	**λ Est**	**F-stat**	**Model P-value**	**Adjusted R^2**	**P-values**					
							Forearm	Diet	Forage Habitat	Forage Mode	Echo Mode	Migrate Type
FA + EM	112.28	0.00	1.00	47.01	*$6.93e^{-11}$*	0.702	*$1.46e^{-11}$*	-	-	-	0.140	-
FA	112.32	0.04	1.00	88.79	*$1.74e^{-11}$*	0.692	*$1.74e^{-11}$*	-	-	-	-	-
FA + FH	112.52	0.24	1.00	32.94	*$2.01e^{-10}$*	0.711	*$1.33e^{-11}$*	-	0.125	-	-	-
FA + FH + EM	113.14	0.86	0.59	26.21	*$4.20e^{-10}$*	0.721	*$2.42e^{-11}$*	-	*0.017*	-	0.071	-
FA + FH + M	113.40	1.11	1.00	26.64	*$9.25e^{-10}$*	0.708	*$2.02e^{-11}$*	-	0.128	-	-	0.422
FA + FH + EM + M	113.63	1.35	0.00	27.29	*$5.39e^{-11}$*	0.771	*$9.71e^{-12}$*	-	0.000	0.061	-	0.157
FA + FM	114.16	1.88	1.00	44.01	*$1.65e^{-10}$*	0.688	*$2.72e^{-11}$*	-	-	0.497	-	-
FA + FM + EM	114.19	1.91	1.00	31.10	*$4.23e^{-10}$*	0.698	*$2.30e^{-11}$*	-	-	0.491	0.141	-
FA + M	114.47	2.19	1.00	43.53	*$1.91e^{-10}$*	0.686	*$3.01e^{-11}$*	-	-	-	-	0.673
FA + FH + FM + EM	114.49	2.21	1.00	20.95	*$1.64e^{-9}$*	0.719	*$1.69e^{-11}$*	-	0.119	0.762	0.095	-
FA + EM + M	114.58	2.29	1.00	30.69	*$5.02e^{-10}$*	0.696	*$2.61e^{-11}$*	-	-	-	0.145	0.687
FA + FH + FM	115.04	2.76	1.00	24.10	*$1.22e^{-9}$*	0.703	*$2.49e^{-11}$*	-	0.132	0.768	-	-
FA + FH + FM + EM + M	115.83	3.54	0.54	18.33	*$3.08e^{-9}$*	0.727	*$3.62e^{-11}$*	-	*0.013*	0.332	0.069	0.184
FA + FM + M	116.47	4.19	1.00	28.72	*$1.17e^{-9}$*	0.681	*$4.80e^{-11}$*	-	-	0.503	-	0.698
FA + FM + EM + M	116.59	4.31	1.00	22.85	*$2.37e^{-9}$*	0.692	*$4.75e^{-11}$*	-	-	0.496	0.145	0.662
FA + FH + FM + M	117.19	4.91	0.64	19.38	*$4.28e^{-9}$*	0.702	*$6.76e^{-11}$*	-	*0.028*	0.428	-	0.249
FA + D + FH + EM	118.59	6.31	0.00	17.86	*$1.27e^{-9}$*	0.776	*$2.12e^{-11}$*	*0.002*	*0.004*	-	*0.031*	-
FA + D	119.37	7.09	1.00	17.76	*$1.23e^{-8}$*	0.682	*$7.76e^{-11}$*	0.596	-	-	-	-
FA + D + EM	119.64	7.36	1.00	15.74	*$1.92e^{-8}$*	0.694	*$6.59e^{-11}$*	0.579	-	-	0.140	-
FA + D + FM + EM	121.01	8.73	1.00	13.89	*$4.12e^{-8}$*	0.698	*$7.59e^{-11}$*	0.573	-	0.698	0.066	-
FA + D + FH	121.37	9.09	0.00	17.42	*$6.52e^{-11}$*	0.747	*$6.52e^{-11}$*	*0.005*	*0.007*	-	-	-
FA + D + FH + FM + EM	121.99	9.71	0.00	15.45	*$5.26e^{-9}$*	0.769	*$4.25e^{-11}$*	*0.003*	*0.005*	0.190	0.078	-
FA + D + FM	122.15	9.87	1.00	14.45	*$5.18e^{-8}$*	0.674	*$1.40e^{-10}$*	0.608	-	0.708	-	-
FA + D + M	122.22	9.94	1.00	14.42	*$5.33e^{-8}$*	0.674	*$1.43e^{-10}$*	0.609	-	-	-	0.774
FA + D + FH + FM	122.62	10.34	0.00	15.77	*$5.68e^{-9}$*	0.752	*$7.05e^{-11}$*	*0.004*	*0.006*	0.206	-	-
FA + D + EM + M	122.71	10.43	1.00	13.12	*$7.86e^{-8}$*	0.685	*$1.26e^{-10}$*	0.593	-	-	0.146	0.805
FA + D + FH + M	122.98	10.69	0.00	15.60	*$6.47e^{-9}$*	0.750	*$7.81e^{-11}$*	*0.004*	*0.006*	-	-	0.248

FA + D + FH + FM + EM + M	123.83	11.55	0.00	14.28	*$1.09e^{-8}$*	0.773	*$5.12e^{-11}$*	*0.003*	*0.004*	0.187	0.076	0.232
FA + D + FM + EM + M	124.27	11.99	1.00	11.81	*$1.57e^{-7}$*	0.689	*$1.47e^{-10}$*	0.587	-	0.702	0.070	0.787
FA + D + FH + FM + M	124.45	12.17	0.00	14.33	*$1.27e^{-8}$*	0.755	*$8.67e^{-11}$*	*0.004*	*0.006*	0.204	-	0.254
FA + D + FM + M	125.20	12.92	1.00	12.05	*$2.02e^{-7}$*	0.665	*$2.61e^{-10}$*	0.621	-	0.712	-	0.787
1	158.19	45.91	0.90	-	-	0.000	-	-	-	-	-	-

Table B. Summary of outputs from phylogenetic generalized least squares regression analysis on principal component (PC) 1 and ecological variables, with forearm (FA) as a size covariate. D: diet, FH: foraging habitat, FM: foraging mode, EM: echolocation mode, M: migratory type.

**Model: PC2**	**AICc**	**ΔAICc**	**λ Est**	**F-stat**	**Model P-value**	**Adjusted R^2**	**P-values**					
							Body Mass	Diet	Forage Habitat	Forage Mode	Echo Mode	Migrate Type
BM + D	90.89	0.00	1.00	3.93	*0.006*	0.273	*0.030*	*0.009*	-	-	-	-
BM + D + FH	91.77	0.88	1.00	3.65	*0.005*	0.322	0.064	*0.006*	0.124	-	-	-
BM + D + EM	92.45	1.56	1.00	3.48	*0.009*	0.277	0.073	*0.009*	-	-	0.287	-
BM + D + FM	93.48	2.59	1.00	3.26	*0.013*	0.258	0.076	*0.010*	-	0.584	-	-
BM + D + M	93.81	2.91	1.00	3.19	*0.014*	0.252	0.077	*0.010*	-	-	-	0.855
BM + D + FH + EM	94.37	3.47	1.00	3.22	*0.009*	0.313	0.066	*0.007*	0.128	-	0.446	-
BM + D + FH + M	95.09	4.19	1.00	3.10	*0.011*	0.331	0.069	*0.008*	0.132	-	-	0.860
BM + D + FH + FM	95.10	4.21	1.00	3.09	*0.011*	0.301	0.069	*0.007*	0.132	0.882	-	-
BM + D + FM + EM	95.33	4.43	1.00	2.95	*0.017*	0.259	0.076	*0.010*	-	0.584	0.312	-
BM + D + EM + M	95.57	4.67	1.00	2.90	*0.018*	0.254	0.077	*0.010*	-	-	0.294	0.880
BM + FH	95.59	4.69	1.00	2.71	0.060	0.116	0.102	-	0.084	-	-	-
BM	96.27	5.37	1.00	2.59	0.116	0.039	0.116	-	-	-	-	-
BM + D + FM + M	96.59	5.70	1.00	2.71	*0.025*	0.235	0.081	*0.012*	-	0.590	-	0.867
1	96.69	5.79	1.00	-	-	-	-	-	-	-	-	-
BM + EM	96.70	5.80	1.00	2.23	0.122	0.059	0.112	-	-	-	0.186	-
BM + FM	97.31	6.41	1.00	1.92	0.162	0.045	0.115	-	-	0.276	-	-
BM + D + FH + FM + EM	97.60	6.71	1.00	2.83	0.016	0.297	0.070	*0.008*	0.134	0.883	0.371	-
BM + FH + EM	97.75	6.86	1.00	2.10	0.102	0.101	0.105	-	0.088	-	0.530	-
BM + FH + M	97.84	6.94	1.00	2.07	0.105	0.099	0.105	-	0.088	-	-	0.571
BM + FH + FM	98.21	7.31	1.00	1.97	0.120	0.091	0.107	-	0.090	0.996	-	-
BM + M	98.58	7.69	1.00	1.28	0.291	0.014	0.121	-	-	-	-	0.871
BM + D + FH + FM + M	98.65	7.75	1.00	1.67	*0.021*	0.278	0.073	*0.009*	0.141	0.884	-	0.865
BM + D + FM + EM + M	98.65	7.76	1.00	2.50	*0.032*	0.236	0.081	*0.012*	-	0.590	0.320	0.881
BM + EM + M	99.12	8.23	1.00	1.46	0.241	0.034	0.117	-	-	-	0.192	0.837
BM + FM + EM	99.15	8.26	1.00	1.45	0.244	0.034	0.117	-	-	0.278	0.455	-
BM + FM + M	99.72	8.83	1.00	1.26	0.302	0.020	0.120	-	-	0.282	-	0.812
BM + FH + FM + EM	100.04	9.15	1.00	1.73	0.154	0.086	0.108	-	0.092	0.996	0.374	-
BM + FH + EM + M	100.16	9.27	1.00	1.71	0.159	0.083	0.109	-	0.092	-	0.534	0.578

BM + FH + FM + M	100.62	9.72	1.00	1.61	0.183	0.073	0.111	-	0.095	0.996	-	0.577
BM + D + FH + FM + EM + M	101.40	10.51	1.00	2.47	*0.028*	0.273	0.075	*0.010*	0.143	0.885	0.379	0.860
BM + FM + EM + M	101.72	10.83	1.00	1.07	0.385	0.007	0.122	-	-	0.285	0.461	0.832
BM + FH + FM + EM + M	102.66	11.77	1.00	1.46	0.222	0.066	0.112	-	0.097	0.997	0.379	0.601

Table C. Summary of outputs from phylogenetic generalized least squares regression analysis on principal component (PC) 2 and ecological variables, with body mass (BM) as a size covariate. D: diet, FH: foraging habitat, FM: foraging mode, EM: echolocation mode, M: migratory type.

Model: PC2	AICc	ΔAICc	λ Est	F-stat	Model P-value	Adjusted R^2	P-values					
							Forearm	Diet	Forage Habitat	Forage Mode	Echo Mode	Migrate Type
FA+ D	98.23	5.39	1	3.42	*0.013*	0.237	0.492	*0.008*	-	-	-	-
FA+ D + EM	98.50	5.66	1	3.06	*0.017*	0.241	0.491	*0.008*	-	-	0.285	-
FA+ D + FM	98.53	5.69	1	2.81	*0.025*	0.218	0.497	*0.009*	-	0.665	-	-
FA+ D + FH	98.62	5.78	1	2.87	*0.019*	0.252	0.488	*0.007*	0.276	-	-	-
FA+ D + M	98.70	5.86	1	2.77	*0.027*	0.214	0.499	*0.009*	-	-	-	0.962
1	99.04	6.20	-	-	-	-	-	-	-	-	-	-
FA+ D + FM + EM	99.07	6.23	1	2.64	*0.029*	0.227	0.495	*0.009*	-	0.663	0.249	-
FA+ D + EM + M	100.37	7.53	1	2.54	*0.034*	0.217	0.498	*0.009*	-	-	0.293	0.942
FA+ D + FH + EM	100.45	7.61	1	2.57	*0.028*	0.244	0.491	*0.008*	0.280	-	0.423	-
FA+ FH	100.57	7.73	1	1.67	0.190	0.049	0.538	-	0.113	-	-	-
FA	100.69	7.85	1	0.36	0.551	-0.017	0.551	-	-	-	-	-
FA+ EM	100.75	7.91	1	1.25	0.297	0.013	0.545	-	-	-	0.152	-
FA+ D + FM + M	100.92	8.09	1	2.34	*0.048*	0.194	0.504	*0.010*	-	0.670	-	0.950
FA+ FM	100.95	8.11	1	1.05	0.361	0.003	0.547	-	-	0.197	-	-
FA+ D + FH + FM	101.00	8.16	1	2.44	*0.036*	0.228	0.495	*0.009*	0.288	0.946	-	-
FA+ D + FM + EM + M	101.05	8.21	1	2.23	0.052	0.202	0.502	*0.010*	-	0.668	0.257	0.957
FA+ FH + EM	101.34	8.50	1	1.39	0.258	0.038	0.540	-	0.116	-	0.446	-
FA+ FH + M	102.69	9.85	1	1.36	*0.027*	0.036	0.541	-	0.117	-	-	0.490
FA+ D + FH + M	103.01	10.17	0.303	4.10	*0.002*	0.388	0.894	*0.001*	0.054	-	-	0.608
FA+ M	103.27	10.43	1	0.23	0.793	-0.041	0.556	-	-	-	-	0.740
FA+ D + FH + FM + EM	103.47	10.63	1	2.34	*0.039*	0.236	0.493	*0.009*	0.284	0.946	0.259	-
FA+ EM + M	104.27	11.43	1	0.86	0.470	-0.011	0.550	-	-	-	0.157	0.717
FA+ FM + EM	105.44	12.60	1	0.85	0.478	-0.012	0.550	-	-	0.200	0.497	-
FA+ FH + FM	105.52	12.68	1	1.24	0.313	0.024	0.543	-	0.120	0.801	-	-
FA+ FM + M	98.23	5.39	1	0.74	0.537	-0.021	0.552	-	-	0.202	-	0.692
FA+ FH + EM + M	98.50	5.66	1	1.19	0.336	0.023	0.543	-	0.120	-	0.450	0.500
FA+ FH + FM + EM	98.53	5.69	1	1.12	0.367	0.016	0.545	-	0.122	0.801	0.407	-
FA+ FH + FM + M	98.62	5.78	1	1.07	0.393	0.001	0.546	-	0.124	0.802	-	0.494

FA+ FM + EM + M	98.70	5.86	1	0.66	0.627	-0.037	0.555	-	-	0.206	0.502	0.709
FA+ D + FH + FM + M	99.04	6.20	0.295	3.56	*0.004*	0.371	0.896	*0.001*	0.057	0.966	-	0.606
FA+ FH + FM + EM + M	99.07	6.23	1	0.99	0.446	-0.001	0.548	-	0.127	0.803	0.411	0.513
FA+ D + FH + FM + EM + M	100.37	7.53	1.00	0.99	0.446	-0.001	0.548	-	0.127	0.803	0.411	0.513

Table D. Summary of outputs from phylogenetic generalized least squares regression analysis on principal component (PC) 2 and ecological variables, with forearm (FA) as a size covariate. D: diet, FH: foraging habitat, FM: foraging mode, EM: echolocation mode, M: migratory type.
