# Supplementary material for: Role of ecology in shaping external nasal morphology in bats and implications for olfactory tracking
Source: PLoS One. 2020 Jan 8;15(1):e0226689. doi: 10.1371/journal.pone.0226689 (PMC6948747; doi:10.1371/journal.pone.0226689)
Supplement: S5 File — Figure A. Boxplots representing variation in phylogenetic Principal Component (PC) 1 (top) and PC 2 (bottom) for species within the species Phyllostomidae across diet categories. Figure B. Boxplots representing variation in phylogenetic Principal Component (PC) 1 (top) and PC 2 (bottom) for species within the species Phyllostomidae across foraging habitat categories. Table A. Summary of outputs from phylogenetic generalized least squares regression analysis on principal components and ecological variables for species within the family Phyllostomidae (n = 22 species), using body mass (BM) as a covariate. Table B. Summary of outputs from phylogenetic generalized least squares regression analysis on principal components and ecological variables for species within the family Phyllostomidae (n = 22 species), using forearm (FA) as a covariate. (PDF) [file pone.0226689.s008.pdf]

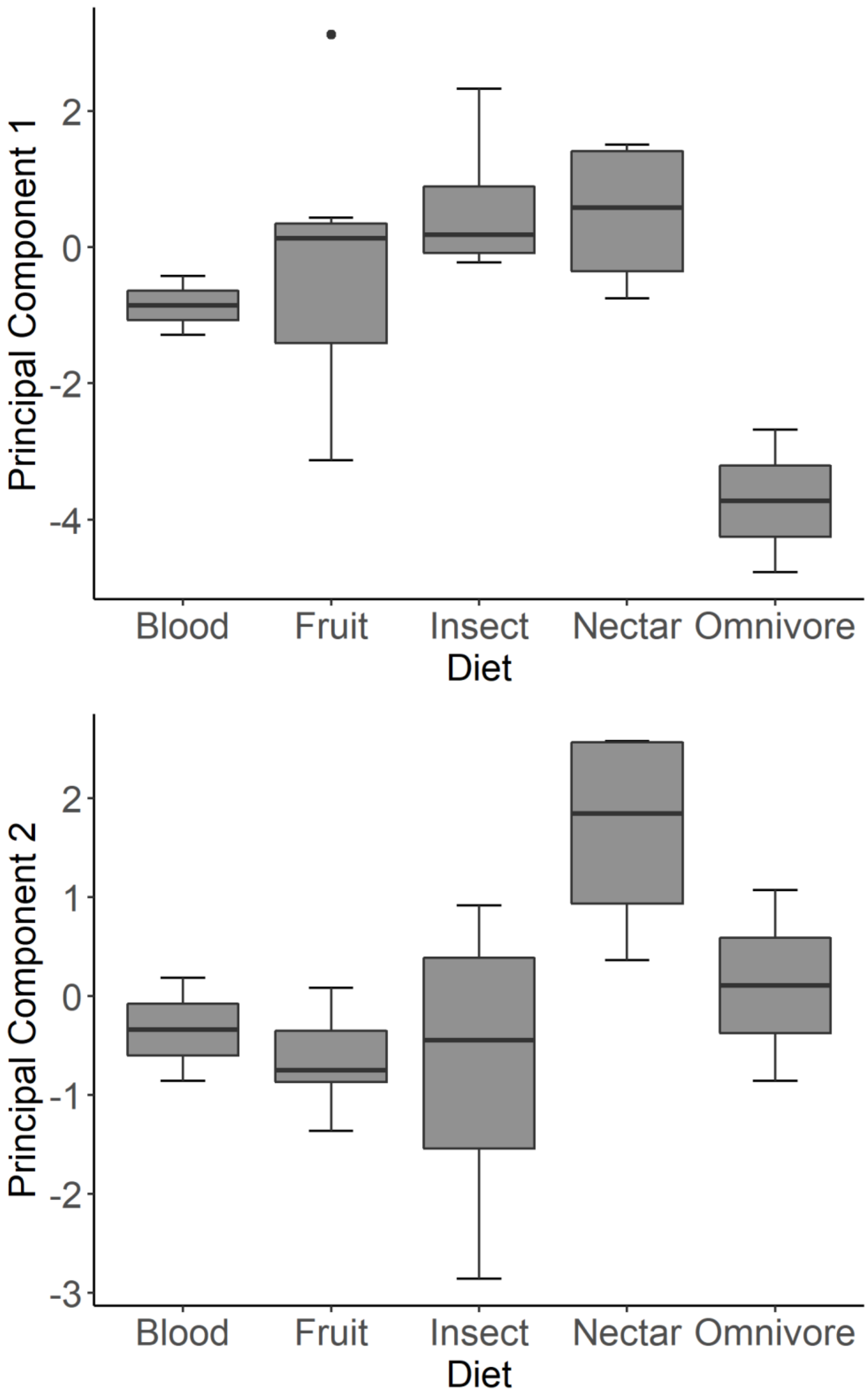

Figure A. Boxplots representing variation in phylogenetic Principal Component (PC) 1 (*top*) and PC 2 (*bottom*) for species within the species Phyllostomidae across diet categories.

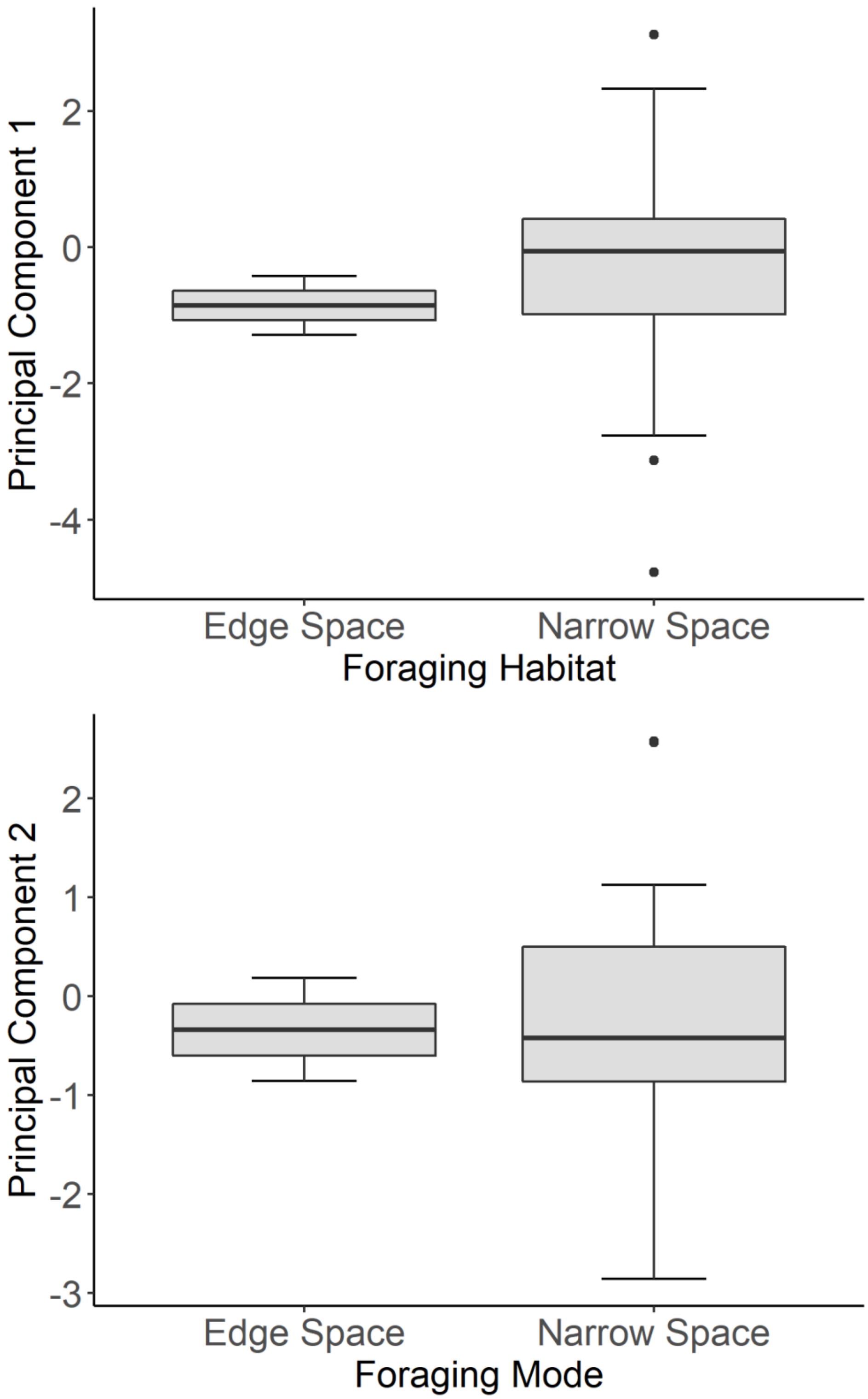

Figure B. Boxplots representing variation in phylogenetic Principal Component (PC) 1 (*top*) and PC 2 (*bottom*) for species within the species Phyllostomidae across foraging habitat categories.

**Models**	**AICc**	**ΔAICc**	**λ Est**	***F*-stat**	**Model *P*-value**	**Adjusted R^2**	**P-values**			
							Body Mass	Diet	Forage Habitat	Migrate Type
**PC1 ~ BM**	64.56	0.00	0.47	54.03	$4.21e^{-7}$	0.716	$4.21e^{-7}$	-	-	-
**PC1 ~ BM + FH**	66.49	1.93	0.00	25.58	$4.07e^{-6}$	0.701	$9.29e^{-7}$	-	0.424	-
**PC1 ~ BM + M**	67.15	2.59	0.43	25.74	$3.90e^{-6}$	0.702	$8.22e^{-7}$	-	-	0.754
**PC1 ~ BM + D**	75.32	10.76	0.00	9.42	*0.0002*	0.6671	$4.77e^{-6}$	0.7918	-	-
**PC1 ~ 1**	90.10	25.54	1.00	-	-	0	$4.21e^{-7}$	-	-	-
**PC2 ~ BM + FH**	68.89	0.00	1.00	2.67	0.095	0.137	0.236	-	0.065	-
**PC2 ~ BM + M**	68.89	0.00	1.00	2.67	0.095	0.137	0.236	-	-	0.065
**PC2 ~ 1**	69.20	0.31	1.00	-	-	0	-	-	-	-
**PC2 ~ BM**	70.23	1.34	1.00	1.32	0.265	0.015	0.265	-	-	-
**PC2 ~ BM + D**	72.60	3.71	1.00	3.53	0.024	0.376	0.694	*0.014*	-	-

Table A. Summary of outputs from phylogenetic generalized least squares regression analysis on principal components and ecological variables for species within the family Phyllostomidae (n = 22 species), using body mass (BM) as a covariate. D: diet, FH: foraging habitat, M: migratory type.

Models	AICc	ΔAICc	λ Est	*F*-stat	Model *P*-value	Adjusted R^2	P-values			
							Body Mass	Diet	Forage Habitat	Migrate Type
**PC1 ~ FA**	54.41	0.00	1.00	105.50	$2.02e^{-9}$	0.833	$2.02e^{-9}$	-	-	-
**PC1 ~ FA + M**	54.76	0.34	1.00	56.86	$9.56e^{-9}$	0.842	$2.17e^{-9}$	-	-	0.159
**PC1 ~ FA + D**	58.87	4.46	0.00	23.45	$7.73e^{-7}$	0.842	$2.65e^{-8}$	*0.017*		
**PC1 ~ FA + FH**	66.49	12.08	0.00	25.58	$4.07e^{-6}$	0.701	$9.29e^{-7}$	-	0.424	-
**PC1 ~ 1**	90.10	25.54	1.00	-	-	0	$4.21e^{-7}$	-	-	-
**PC2 ~ 1**	69.20	0.00	1.00	-	-	0	-	-	-	-
**PC2 ~ FA + M**	69.93	0.73	1.00	2.11	0.144	0.095	0.639	-		0.061
**PC2 ~ FA**	71.42	2.22	1.00	0.20	0.661	-0.040	0.661	-	-	-
**PC2 ~ FA + D**	73.11	3.91	0.00	3.37	*0.029*	0.361	0.805	*0.016*	-	-
**PC2 ~ FA + FH**	73.73	4.53	1.00	0.26	0.771	-0.075	0.667	-	0.569	-

Table B. Summary of outputs from phylogenetic generalized least squares regression analysis on principal components and ecological variables for species within the family Phyllostomidae (n = 22 species), using forearm (FA) as a covariate. D: diet, FH: foraging habitat, M: migratory type.
