# Supplementary material for: Role of ecology in shaping external nasal morphology in bats and implications for olfactory tracking
Source: PLoS One. 2020 Jan 8;15(1):e0226689. doi: 10.1371/journal.pone.0226689 (PMC6948747; doi:10.1371/journal.pone.0226689)
Supplement: S6 File — Figure A. Phylogenetic principal component (PC) 1 (top) and PC2 (bottom) plotted against log average speed for a subset of dataset (n = 21 species). Figure B. Phylogenetic principal component (PC) 1 (top) and PC2 (bottom) plotted against log wing loading for a subset of dataset (n = 25 species). Figure C. Phylogenetic principal component (PC) 1 (top) and PC2 (bottom) plotted against log aspect ratio for a subset of dataset (n = 35 species). Table A. Summary of outputs from phylogenetic generalized least squares regression analysis on principal components and log flight speed (n = 21 species). Table B. Summary of outputs from phylogenetic generalized least squares regression analysis on principal components and log wing loading (n = 25 species). Table C. Summary of outputs from phylogenetic generalized least squares regression analysis on principal components and log aspect ratio (n = 35 species). (PDF) [file pone.0226689.s009.pdf]

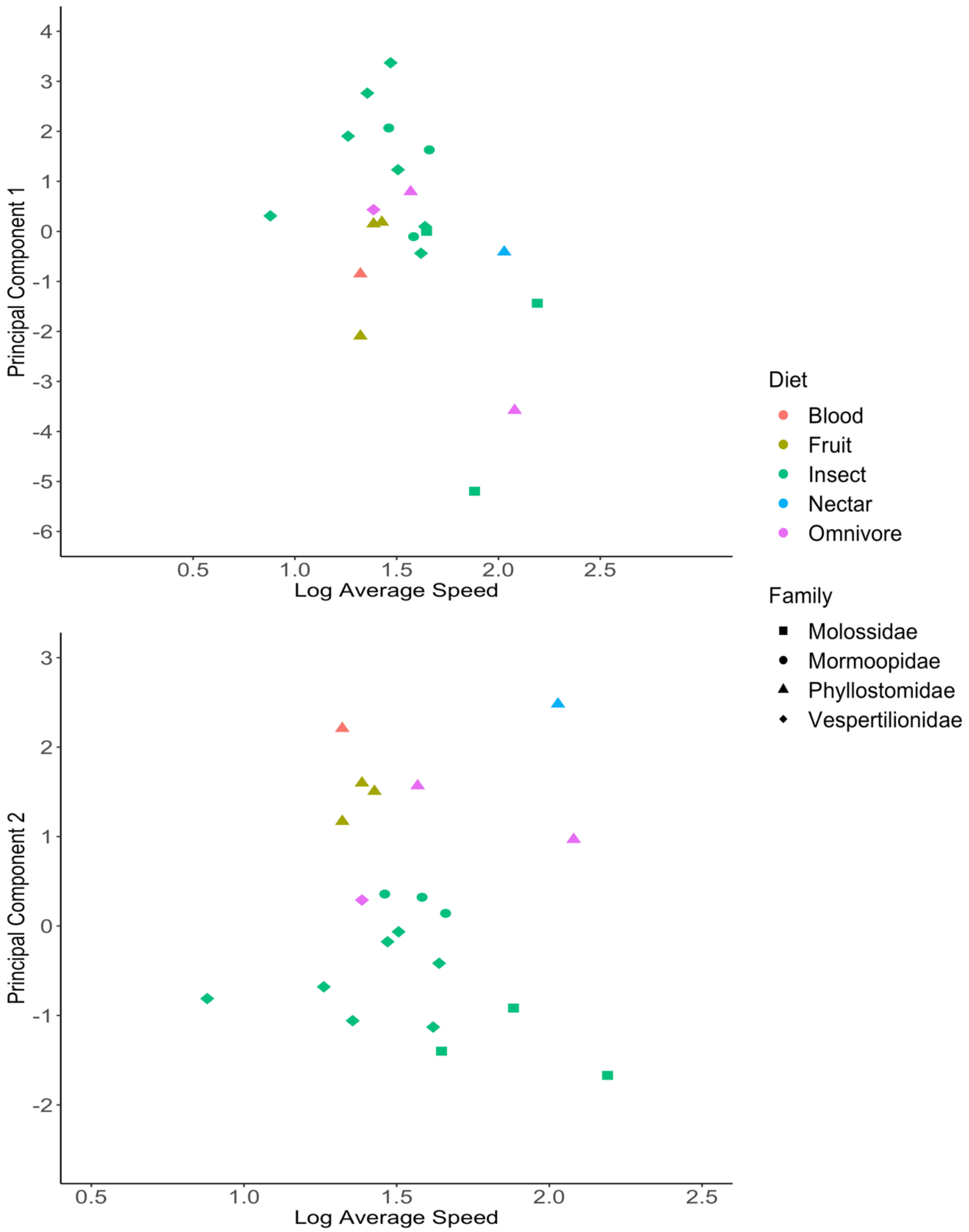

Figure A. Phylogenetic principal component (PC) 1 (*top)* and PC2 (*bottom*) plotted against log average speed for a subset of dataset (n = 21 species).

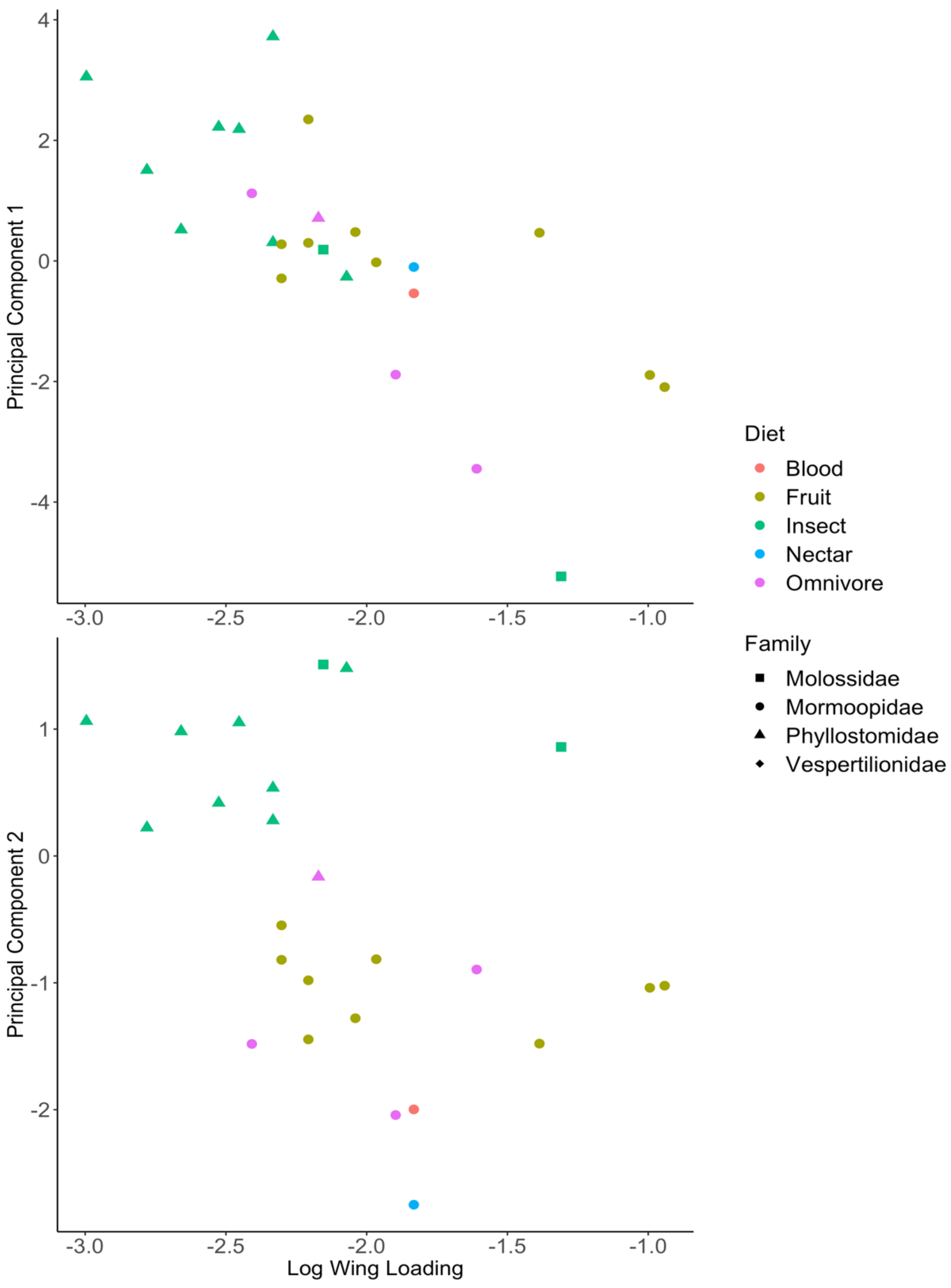

Figure B. Phylogenetic principal component (PC) 1 (*top)* and PC2 (*bottom*) plotted against log wing loading for a subset of dataset (n = 25 species).

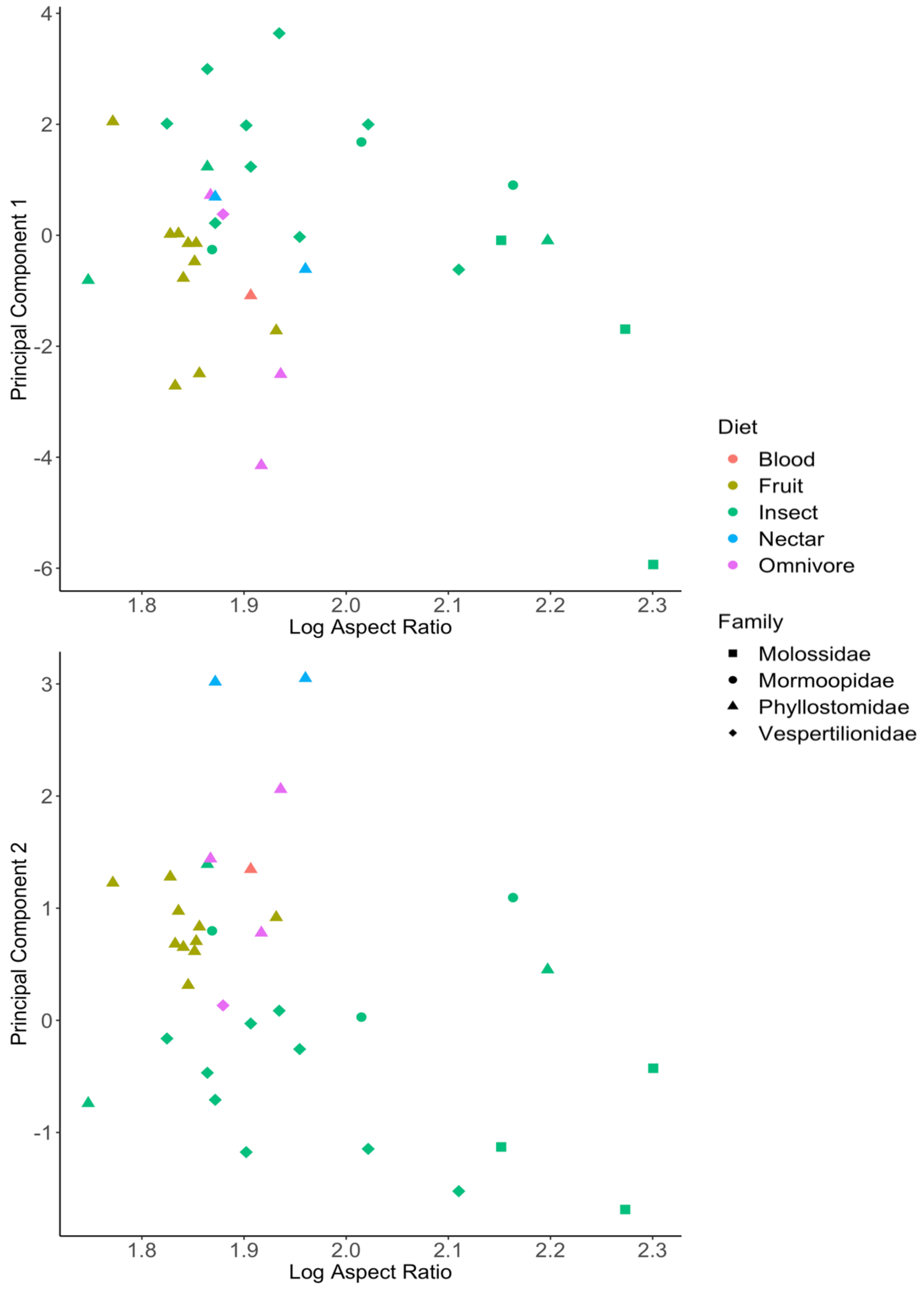

Figure C. Phylogenetic principal component (PC) 1 (*top)* and PC2 (*bottom*) plotted against log aspect ratio for a subset of dataset (n = 35 species).

Table A. Summary of outputs from phylogenetic generalized least squares regression analysis on principal components and log flight speed (n = 21 species). BM: body mass, FA: forearm, S: speed (log transformed), D: diet.

**Flight Speed**	**AICc**	**ΔAICc**	**λ Est**	**F-stat**	**Model P-value**	**Adjusted R^2**	**P-value**		
							Body Mass	Log Speed	Diet
**PC1 ~ BM**	66.15	0	0.45	42.33	*$3.12e^{-6}$*	0.674	*$3.12e^{-6}$*	-	-
**PC1 ~ BM + S**	68.67	2.52	0.63	20.30	*$2.44e^{-5}$*	0.659	*$5.61e^{-6}$*	0.560	-
**PC1 ~ BM + S + D**	79.76	13.61	1.00	7.348	0.001	0.656	$2.26e^{-5}$	0.193	0.488
**PC1 ~ 1**	88.25	22.09	0.58	-	-	0.000	-	-	-
**PC2 ~ BM + S + D**	51.71	1.58	0.67	4.193	*0.013*	0.489	0.348104	0.362	0.006
**PC2 ~ 1**	50.59	0.46	0.95	-	-	0.000	-	-	-
**PC2 ~ BM**	52.26	2.13	0.97	0.77	0.390	-0.011	0.390	-	-
**PC2 ~ BM + S**	54.81	4.68	0.97	0.44	0.651	-0.059	0.411	0.687	-
								$1.71e^{-5}$	
							Forearm	Log Speed	Diet
**PC1 ~ FA**	66.89	0	0.57	40.050	*$4.50e^{-6}$*	0.661	*$4.50e^{-6}$*	-	-
**PC1 ~ FA + S**	67.81	0.92	1.00	22.990	*$1.10e^{-5}$*	0.687	*$3.56e^{-6}$*	0.113	-
**PC1 ~ FA + S + D**	78.97	12.08	1.00	7.719	*0.001*	0.668		0.128	0.579
**PC1 ~ 1**	88.25	21.36	0.58	-	-	0.000	-	-	-
**PC2 ~ FA + S + D**	50.24	0.00	0.69	4.628	*0.008*	0.521	0.76348	0.553	0.003
**PC2 ~ 1**	50.59	0.35	0.95	-	-	0.000	-	-	-
**PC2 ~ FA**	52.70	2.47	0.97	0.350	0.561	-0.034	0.561	-	-
**PC2 ~ FA + S**	55.37	5.13	0.97	0.194	0.825	-0.088	0.578	0.798	-

Table B. Summary of outputs from phylogenetic generalized least squares regression analysis on principal components and log wing loading (n = 25 species). BM: body mass, FA: forearm, WL: wing loading (log transformed), D: diet.

**Wing Loading**	**AICc**	**ΔAICc**	**λ Est**	**F-stat**	**Model P-value**	**Adjusted R^2**	**P-value**		
							Body Mass	Wing Loading	Diet
**PC1 ~ BM**	76.16	0.00	0.44	53.280	*$1.991e^{-7}$*	0.685	*$1.99e^{-7}$*	-	-
**PC1 ~ BM + WL**	78.22	2.07	0.43	26.330	*$1.454\ e^{-6}$*	0.679	*$3.08e^{-7}$*	0.499	-
**PC1 ~ BM + WL + D**	89.27	13.11	0.00	9.377	9.71E-05	0.677	8.02E-07	0.539	0.762
**PC1 ~ 1**	102.42	26.27	1.00	-	-	0	-	-	-
**PC2 ~ BM + WL + D**	51.54	4.05	0.00	17.260	1.42E-06	0.803	0.000601	0.051	1.67E-06
**PC2 ~ 1**	54.78	7.28	1.00	-	-	0	-	-	-
**PC2 ~ BM**	55.95	8.45	1.00	1.133	0.298	0.006	0.298	-	-
**PC2 ~ BM + WL**	58.22	10.73	1.00	0.692	0.511	-0.026	0.306	0.599	-
							Forearm	Wing Loading	Diet
**PC1 ~ FA**	70.06	0.00	0.65	72.00	*$1.54e^{-8}$*	0.747	*$1.54e^{-8}$*	-	-
**PC1 ~ FA + WL**	72.13	2.07	1.00	36.44	*$1.04e^{-7}$*	0.747	*$2.12e^{-8}$*	0.441	-
**PC1 ~ FA + WL + D**	81.87	11.81	0.00	13.640	*$7.76e^{-6}$*	0.760	*$7.02e^{-8}$*	0.114	0.565
**PC1 ~ 1**	102.42	32.37	1.00	-	-	0	-	-	-
**PC2 ~ FA + WL + D**	52.26	4.29	0.00	16.680	*$1.82e^{-6}$*	0.797	*0.047*	*$4.30e^{-4}$*	*$2.60e^{-6}$*
**PC2 ~ 1**	54.78	6.81	1.00	-	-	0	-	-	-
**PC2 ~ FA**	55.57	7.60	1.00	1.49	0.234	0.020	0.234	-	-
**PC2 ~ FA + WL**	58.01	10.04	1.00	0.79	0.466	-0.018	0.243	0.710	-

Table C. Summary of outputs from phylogenetic generalized least squares regression analysis on principal components and log aspect ratio (n = 35 species). BM: body mass, FA: forearm, AR: aspect ratio (log transformed), D: diet.

**Aspect Ratio**	**AICc**	**ΔAICc**	**λ Est**	**F-stat**	**Model P-value**	**Adjusted R^2**	**P-value** Body Mass	Aspect Ratio	Diet
**PC1 ~ BM**	109.72	0.00	0.72	52.57	*$2.57e^{-8}$*	0.603	*$2.57e^{-8}$*	-	-
**PC1 ~ BM + AR**	111.64	1.92	0.70	26.15	*$1.861e^{-7}$*	0.597	*$3.54e^{-8}$*	-	-
**PC1 ~ BM + AR + D**	122.22	12.50	0.75	7.848	*$5.14e^{-5}$*	0.547	*$2.26e^{-7}$*	0.542	0.954
**PC1 ~ 1**	140.24	30.52	1.00	-	-	-	-	-	-
**PC2 ~ BM + AR + D**	79.19	7.89	1.00	3.557	*0.009*	0.311	0.055	0.582	*0.008*
**PC2 ~ BM**	82.30	11.00	1.00	2.93	0.096	0.054	0.096	-	-
**PC2 ~ 1**	82.99	11.69	1.00	-	-	-	-	-	-
**PC2 ~ BM + AR**	84.43	13.13	1.00	1.54	0.230	0.031	0.101	0.642	-
							Forearm	Aspect Ratio	Diet
**PC1 ~ FA**	99.86	0.00	1.00	78.56	*$3.04e^{-10}$*	0.695	*$3.04e^{-10}$*	-	-
**PC1 ~ FA + AR**	102.25	2.39	1.00	38.09	*$3.43e^{-9}$*	0.686	*5.66^{-10}*	0.955	-
**PC1 ~ FA + AR + D**	111.35	11.49	1.00	12.170	1.04E-06	0.664	3.58E-09	0.957	0.757
**PC1 ~ 1**	140.24	40.38	1.00	-	-	-	-	-	-
**PC2 ~ FA + AR + D**	79.97	8.47	1.00	3.376	*0.012*	0.295	0.322	0.876	*0.004*
**PC2 ~ 1**	82.99	11.49	1.00	-	-	-	-	-	-
**PC2 ~ FA**	84.50	13.00	1.00	0.71	0.405	-0.009	0.405	-	-
**PC2 ~ FA + AR**	86.88	15.38	1.00	0.35	0.705	-0.040	0.413	0.898	-
